# LEDGF Binds H3R17me2a Promoting De Novo Nucleotide Biosynthesis in *SETD2* Mutant Clear Cell Renal Cell Carcinoma

**DOI:** 10.1002/advs.202416809

**Published:** 2025-06-23

**Authors:** Yuwei Zhang, Yuhua Zhou, Yuezhou Zhang, Jing Lv, Yang Shen, Dong Zhang, Bo Liu, Wei Zhao, Junyi Ju, Qingyi Zhu, Ke Wang, Ninghan Feng

**Affiliations:** ^1^ Medical School of Nantong University 9 Qiangyuan Road Nantong 226019 China; ^2^ Department of Urology Jiangnan University Medical Center 68 Zhongshan Road Wuxi 214002 China; ^3^ Wuxi School of Medicine Jiangnan University 1800 Lihu Avenue Wuxi 214122 China; ^4^ School of Clinical Medicine The First Affiliated Hospital Chengdu Medical College 783 Xindu Avenue Chengdu 610500 China; ^5^ The Affiliated Taizhou People's Hospital Taizhou School of Clinical Medicine Nanjing Medical University 101 Longmian Avenue Nanjing 211166 China; ^6^ Department of Urology The Second Affiliated Hospital of Nanjing Medical University 121 Jiangjiayuan Nanjing 210003 China; ^7^ Department of Urology The Affiliated Hospital of Qingdao University 1677 Wutaishan Road Qingdao 266001 China

**Keywords:** clear cell renal cell carcinoma, de novo nucleotide biosynthesis pathway, H3R17me2a, LEDGF, SETD2

## Abstract

Previous studies have identified that lens epithelium‐derived growth factor (LEDGF) interacts with SETD2‐dependent histone H3 trimethylated at lysine 36 (H3K36me3) to mediate transcriptional elongation. However, the original LEDGF recognition H3K36me3 epigenetic regulatory axis no longer exists in *SETD2* mutant clear cell renal cell carcinoma (ccRCC) patients, and a new transcription system needs to be discovered. In this study, the authors demonstrated the novel interaction between LEDGF and H3R17me2a. In detail, Asn38 and Asp57 of LEDGF Proline‐Tryptophan‐Tryptophan‐Proline (PWWP) domain are the key binding sites validated by peptide pull‐down assays. Subsequently, a series of in vitro and in vivo experiments showed that *PPAT*, *PAICS*, *GART*, *ADSL*, and *ADSS2* are key target genes. Collectively, LEDGF binds H3R17me2a to regulate purine nucleotide metabolism in *SETD2* mutant ccRCC cells, promoting tumor proliferation, and may be an effective therapeutic target.

## Graphical abstract

In *SETD2*‐mutant ccRCC, the original LEDGF recognition of the H3K36me3 regulatory axis no longer exists. LEDGF interacts with CARM1‐dependent H3R17me2a regulating the transcription of key enzymes in the de novo synthesis pathway. The abnormally elevated LEDGF leads to the accumulation of purine nucleotides in *SETD2*‐mutant ccRCC, thereby satisfying the requirements for the extensive proliferation of malignant tumor cells.

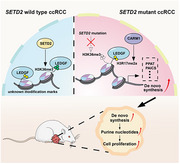

## 1 Introduction

Renal cell carcinoma (RCC) is a common malignancy of the urinary system, with an estimated 400000 new cases worldwide in 2022, and the incidence is still rising.^[^ ^1^ ^]^ The majority of patients with RCC are found by chance on abdominal imaging, and more than half are in stage I at diagnosis.^[^ ^2^ ^]^ However, due to individual patient differences, including tumor size, location, and genetic background, it remains unclear which patients benefit from the specific treatment.^[^ ^3^ ^]^ It is urgent to study the subtype classification of RCC.

Studies have illustrated that tumors can reprogram pathways related to nutrient absorption and metabolism to support the needs of cancer cells for excessive proliferation.^[^ ^4^ ^]^ This phenomenon is known as metabolic reprogramming, and it is promoted by the loss of tumor suppressor genes and the activation of oncogenes.^[^ ^5^ ^]^ RCC includes more than 10 subtypes of histology and molecular pathology, of which clear cell renal cell carcinoma (ccRCC) is the most common and accounts for most cancer‐related deaths.^[^ ^6^ ^]^ ccRCC, sometimes referred to as a “metabolic disease”, has dramatic changes in lipid metabolism and other pathways.^[^ ^7^ ^]^ The metabolic reprogramming promotes rapid proliferation of ccRCC cells, leading to poor prognosis. New therapies targeting key proteins or enzymes involved in dysregulated metabolic pathways in ccRCC are being developed.^[^ ^8^, ^9^, ^10^ ^]^

Molecular analysis of ccRCC has shown that this complex and deadly disease is caused by high‐frequency mutations of multiple tumor suppressor genes such as *VHL*, *PBRM1*, *SETD2*, and *BAP1*, leading to genomic instability and metabolic reprogramming.^[^ ^11^, ^12^, ^13^ ^]^ *SETD2* is one of the most frequently mutated chromatin modification genes across cancers.^[^ ^14^, ^15^ ^]^ Based on the Cancer Genome Atlas (TCGA) database, *SETD2* mutates in 13% of ccRCC patients, 9% of lung adenocarcinoma patients, 7% of bladder urothelial carcinoma patients, and 5% of hepatocellular carcinoma patients.^[^ ^16^ ^]^ In addition, we found that SETD2 expression was significantly down‐regulated in ccRCC, and either low SETD2 expression or high frequency mutations were associated with poor prognosis. In fact, it has been reported that SETD2 catalyses H3K36me3, a classic tumor suppressor marker in ccRCC, participating in, for instance, transcriptional elongation and transcriptional initiation inhibition.^[^ ^17^ ^]^ *SETD2* mutation induces epigenetic remodeling and promotes the malignant progression of ccRCC, but the specific mechanism remains unclear.

However, in *SETD2* mutant ccRCC, the original regulatory axis (Lens Epithelium‐Derived Growth Factor (LEDGF) reads H3K36me3) is no longer present, and new transcriptional recognition system remains to be discovered. LEDGF regulates gene expression and is involved in vital biological processes such as DNA damage repair, playing an indispensable role in maintaining genome stability.^[^ ^18^ ^]^ Previous studies focused on the role of LEDGF in Human Immunodeficiency Virus (HIV) infection and Mixed Lineage Leukemia gene (MLL) leukemia,^[^ ^19^, ^20^ ^]^ overlooking its vital functions in solid tumors. In fact, LEDGF is dysregulated in a variety of solid tumors and is overexpressed in ccRCC.^[^ ^16^ ^]^ The LEDGF Proline‐Tryptophan‐Tryptophan‐Proline (PWWP) domain is reported as a member of the “Royal” family, and is the classic reader of H3K36me3.^[^ ^21^ ^]^ As a vital chromatin localization protein, LEDGF acts as a molecular bridge. The LEDGF PWWP domain and Integrase Binding Domain (IBD) perform the functions of chromatin recognition and protein binding, respectively.^[^ ^22^ ^]^ In detail, it involves processes such as chromatin and DNA binding, transcriptional regulation, and protein‐protein interactions.^[^ ^23^ ^]^ LEDGF may bind to other modified sites on histones, and this novel epigenetic axis may mediate metabolic reprogramming of *SETD2* mutant ccRCC, leading to poor prognosis.

In this study, we identified the novel interaction between LEDGF and H3R17me2a. In detail, Asn38 and Asp57 of LEDGF PWWP domain are the key binding sites validated by peptide pull‐down assays. Subsequently, a series of in vitro and in vivo experiments revealed that LEDGF binds H3R17me2a to regulate purine nucleotide metabolism in *SETD2* mutant ccRCC cells, promoting tumor proliferation, and may be an effective therapeutic target.

## 2 Results

### 2.1 LEDGF is Closely Associated with Tumor and is Highly Expressed in ccRCC

LEDGF has the PWWP domain (Figure S1A, Supporting Information), an important member of “Royal” family, which is reported to recognize methylation modifications at histone lysine sites such as H3K36me3. Protein‐protein interaction (PPI) network analysis showed that LEDGF can directly bind to histone H3 and some histone modification enzymes (Figure S1B,C, Supporting Information), suggesting that LEDGF has the potential to recognize other histone modification sites as an important epigenetic regulatory molecule. Although LEDGF is strongly associated with tumors (Figure S1D, Supporting Information) from the canSAR database, previous studies have highlighted its key roles in MLL leukemia with few studies related to ccRCC.

We explored LEDGF expression across cancers (Figure S1E,F, Supporting Information) on GEPIA database and UALCAN database. LEDGF showed different expression patterns in different tumors, suggesting that LEDGF has certain tissue specificity. Interestingly, although there was no statistical difference in LEDGF mRNA expression levels between ccRCC tissues and normal tissues, LEDGF protein level was significantly higher in ccRCC (Figure S1G, Supporting Information).

### 2.2 High Frequency of *SETD2* Mutation is Associated with Poor Prognosis in ccRCC Patients

LEDGF has been reported to read SETD2‐dependent H3K36me3 mediating transcriptional elongation (Figure S2A, Supporting Information). We searched SETD2 expression in ccRCC on GEPIA database. The results showed that SETD2 was significantly down‐regulated in ccRCC compared to normal tissues (Figure S2B, Supporting Information). The Kaplan‐Meier (K‐M) survival curve from the GEPIA database showed that low expression of SETD2 was associated with worse prognosis in ccRCC patients (Figure S2C, Supporting Information).

We also conducted an online analysis of *SETD2* mutation in 830 ccRCC samples from 5 independent datasets on the cBioPortal database. The results showed that *SETD2* has mutations such as truncation mutation, deep deletion or missense mutation in up to 13% of ccRCC samples (Figure S2D, Supporting Information). This will eventually lead to a loss of function of SETD2, resulting in a series of pathological changes. We analyzed the *SETD2* mutation in patients with different Tumor, Node, and Metastasis Stages (TNM stage) (Figure S2E, Supporting Information). The results showed that compared with patients without *SETD2* mutation, a higher proportion of mutated‐*SETD2* patients had advanced T stages (T3 and T4). In addition, ccRCC patients with *SETD2* mutations have shorter disease‐free and progression‐free survival (Figure S2F,G, Supporting Information).

### 2.3 LEDGF Specifically Binds to H3R17me2a

The mechanism by which *SETD2* mutation leads to poor outcomes in ccRCC patients has not been elucidated. In fact, in *SETD2* mutant patients, the original LEDGF‐H3K36me3 regulatory axis is no longer present. However, LEDGF, as a classic chromatin binding protein, may recognize other histone modification marks to regulate tumor progression.

**Figure 1:**
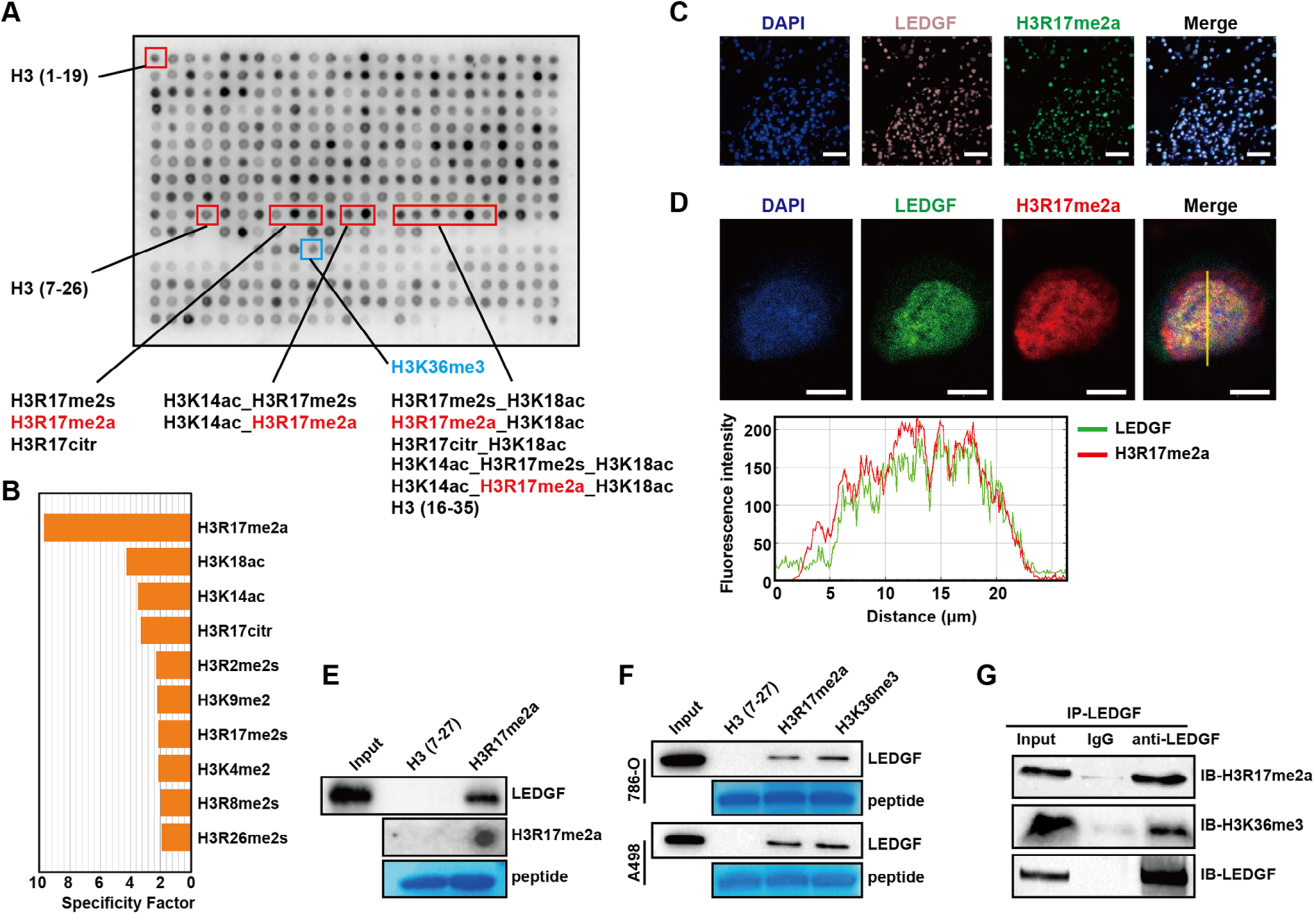
LEDGF specially binds H3R17me2a. A) LEDGF protein was incubated with modified Histone Peptide Array (Active Motif, 13 005). Red and blue boxes indicate specific modification marks. B) The strength of dots was measured using the Array Analyze Software (Active Motif), and the top ten modification marks with the strongest binding ability to LEDGF were showed. C) Immunofluorescence staining of LEDGF (pink) and H3R17me2a (green) in ccRCC tissues. Scale bar = 50 µm. D) Confocal microscopy images of immunofluorescence staining showing that LEDGF (green) colocalizes with H3R17me2a (red) in A498 cells. Scale bar = 5 µm. E) Peptide pull‐down assay was used to detect the interactions between H3 peptide, H3R17me2a peptide, and LEDGF. Dot blot experiment was used to validate the synthesized H3R17me2a peptide. Coomassie Blue staining represents H3 and H3R17me2a peptide. F) Peptide pull‐down assay was used to detect the interactions between H3 peptide, H3R17me2a peptide, H3K36me3 peptide, and LEDGF in ccRCC cells. G) Co‐IP experiment was performed to detect the interactions between H3R17me2a, H3K36me3, and LEDGF in A498 cells.

We first obtained purified LEDGF protein (Figure S3A, Supporting Information). To explore novel modification marks recognized by LEDGF, we incubated LEDGF protein with modified Histone Peptide Array (**Figure** **1**A). The quality control results showed high reliability of the experiment (Figure S3B,C, Supporting Information). Results showed that LEDGF had the strongest binding capacity with H3R17me2a (Figure 1B), which has not been reported yet. The repeated results were consistent with the previous experiment, demonstrating the reliability and stability of LEDGF binding to H3R17me2a (Figure S3D, Supporting Information).

In order to explore the combination of the two in ccRCC, we performed Immunofluorescence experiments in ccRCC tissues and cells. The results showed that LEDGF and H3R17me2a were highly colocalized in the nucleus in both ccRCC tissues (Figure 1C) and ccRCC cells (A498 in Figure 1D; and 786‐O in Figure S3E, Supporting Information). However, we noted that the purified LEDGF protein appeared to bind poorly to H3K36me3 on the modified Histone Peptide array, contrary to previous studies. We synthesized biotinylated H3 peptide, H3R17me2a peptide and H3K36me3 peptide for further verification. The peptide pull‐down assay suggested that H3R17me2a could stably bind to LEDGF, whether it was purified protein (Figure 1E) or from cell lysate (Figure 1F). In addition, we found that LEDGF binds to both H3R17me2a and H3K36me3 in ccRCC cells. The Co‐Immunoprecipitation (Co‐IP) assay also demonstrated the stable binding of LEDGF to H3R17me2a and H3K36me3 in A498 cells (Figure 1G).

### 2.4 LEDGF Binds to H3R17me2a Depending on the Asn38 and Asp57

**Figure 2:**
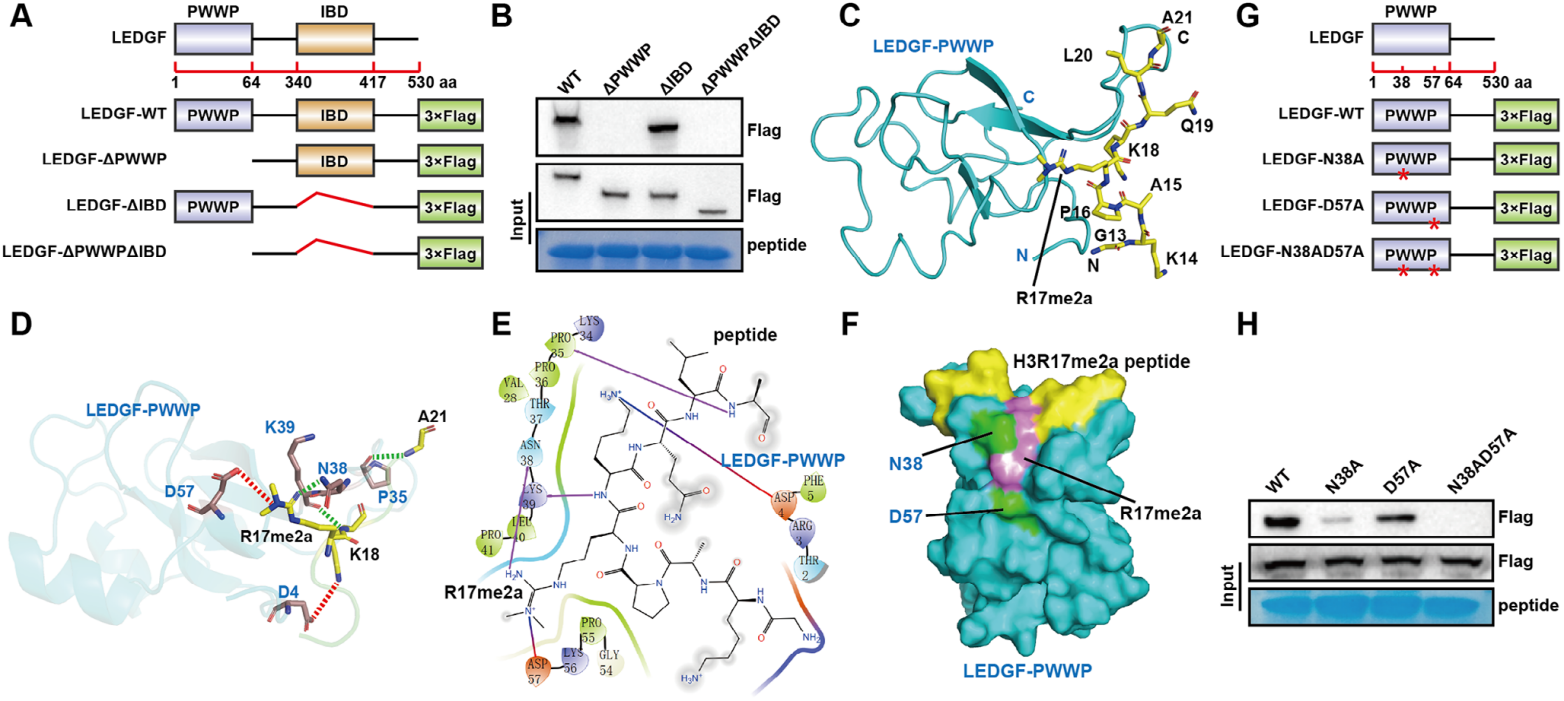
Specific sites of LEDGF facilitate binding with H3R17me2a. A) Schematic representation of LEDGF‐WT, LEDGF‐ΔPWWP, LEDGF‐ΔIBD, and LEDGF‐ΔPWWPΔIBD plasmids. Δ means deletion. B) Peptide pull‐down assay was used to detect the interactions between LEDGF‐WT, LEDGF‐ΔPWWP, LEDGF‐ΔIBD, LEDGF‐ΔPWWPΔIBD, and H3R17me2a peptide. C) Overall structure of LEDGF‐PWWP (left) bound to H3R17me2a peptide (right). Carbon atoms are shown in yellow, nitrogen atoms in blue, and oxygen atoms in red. D) Close‐up view of LEDGF‐PWWP combined with H3R17me2a. LEDGF‐PWWP and the peptide interaction residues are shown in dashed lines. The dashed green lines represent hydrogen bonds, and the dashed red lines represent salt Bridges. E) Schematic of the LEDGF‐PWWP (colorful) and H3R17me2a (black) interactions. Hydrogen bonds and salt bridges are shown as purple and gradient lines, respectively. F) The surface diagram showed that H3R17me2a was well embedded into LEDGF‐PWWP. G) Schematic representation of LEDGF‐WT, LEDGF‐N38A, LEDGF‐D57A, and LEDGF‐N38AD57A plasmids. H) Peptide pull‐down assay was used to detect the interactions between LEDGF^WT^, LEDGF^N38A^, LEDGF^D57A^, LEDGF^N38AD57A^, and H3R17me2a peptide.

To delineate the functional significance of the LEDGF PWWP and IBD domains in mediating LEDGF's recognition of H3R17me2a, we constructed a series of Flag‐tagged plasmids, including LEDGF‐WT (wild type), LEDGF‐ΔPWWP, LEDGF‐ΔIBD, and LEDGF‐ΔPWWPΔIBD (dual deletion) (**Figure** **2**A). Peptide pull‐down assay verified that LEDGF PWWP domain plays a vital role in the binding relationship between LEDGF and H3R17me2a (Figure 2B).

We simulated the interaction of LEDGF with H3R17me2a (Figure 2C). The results showed that there might be three hydrogen bonds and two salt bridges in the interacting residues, and Asn38 and Asp57 of LEDGF directly bind to R17me2a site (Figure 2D,E). The surface diagram showed that H3R17me2a was well embedded in the LEDGF PWWP domain (Figure 2F).

To further clarify the key sites of LEDGF's recognition of H3R17me2a, we constructed a series of Flag‐tagged point mutation plasmids including LEDGF‐N38A, LEDGF‐D57A, and LEDGF‐N38AD57A (Figure 2G). Finally, we performed peptide pull‐down assays with biotinylated H3R17me2a peptide in different groups of cells. The results showed that H3R17me2a peptide had weakened binding ability to LEDGF^D57A^, and almost did not bind to LEDGF^N38A^ or LEDGF^N38AD57A^ (Figure 2H). Our results demonstrate that LEDGF binds to H3R17me2a depending on Asn38 and Asp57.

### 2.5 LEDGF is Overexpressed in ccRCC and Promotes Proliferation

**Figure 3:**
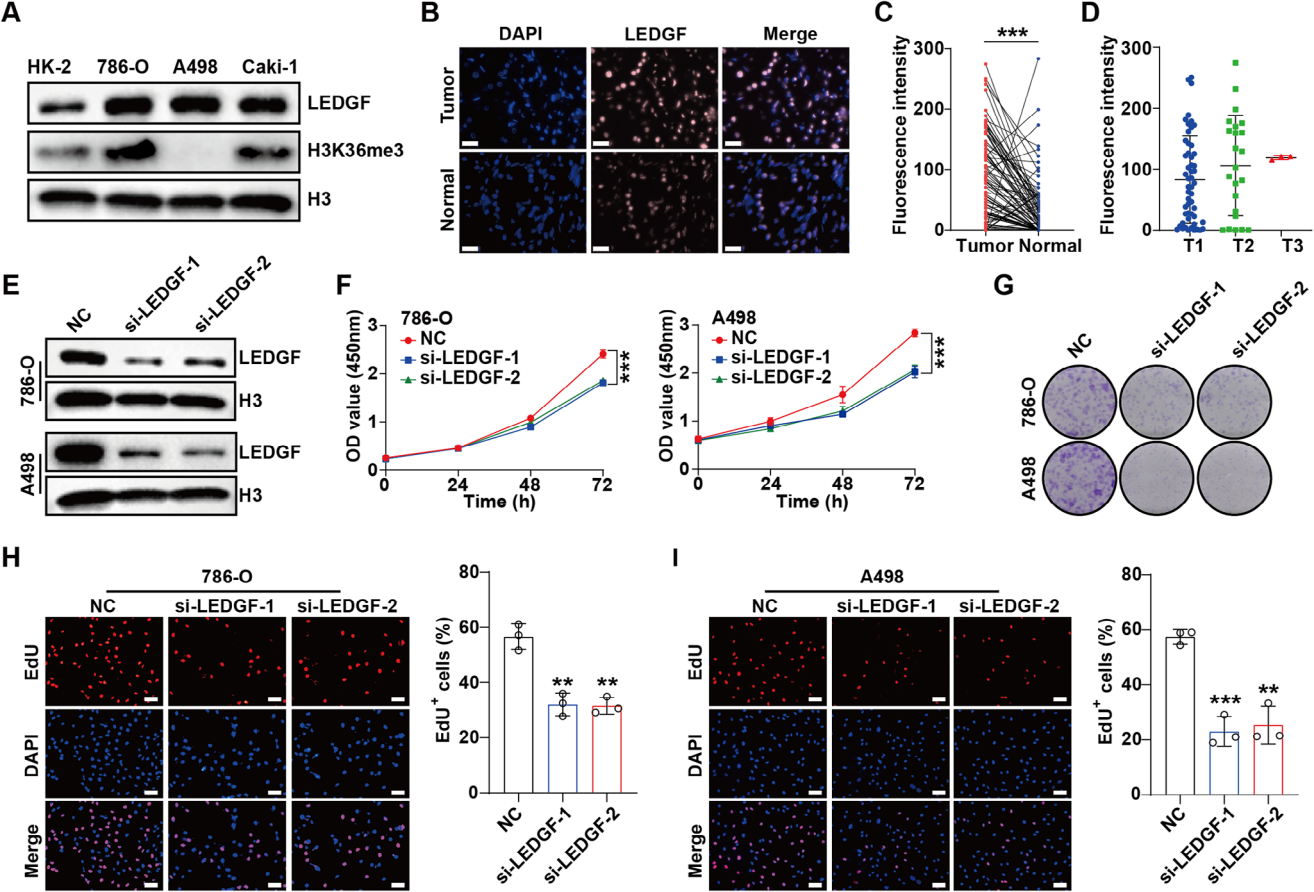
LEDGF is highly expressed in ccRCC and promotes proliferation. A) LEDGF is highly expressed in ccRCC cells. A498 is a *SETD2*‐mutant cell line while 786‐O and Caki‐1 are *SETD2*‐WT cell lines. B,C) Immunofluorescence staining using the tissue microarray revealed that LEDGF is highly expressed in ccRCC tissue samples. Scale bar = 100 µm. (Tumor n = 79, Normal n = 79) D) CcRCC patients with advanced T stage have higher LEDGF expression, which may be associated with poor prognosis. (T1 n = 53, T2 n = 23, T3 n = 3) E) The siRNA targeting LEDGF mRNA can effectively interfere with LEDGF expression in ccRCC cells. F–I) LEDGF knockdown significantly inhibited the proliferation of ccRCC cells. CCK‐8 assay (F), colony formation assay (G), and EdU proliferation assay H,I) were performed to detect the proliferation ability of ccRCC cells after LEDGF knockdown. Scale bar = 100 µm. Data are shown as mean ± SD. **p < 0.01, ***p < 0.001.

To explore the role of LEDGF, we first evaluated LEDGF expression in ccRCC. The results showed that LEDGF was significantly up‐regulated in all three ccRCC cells compared with HK‐2 cells (**Figure** **3**A). In addition, results of the tissue microarray indicated that LEDGF was also overexpressed in ccRCC tissues compared with adjacent normal tissues (Figure 3B,C), and higher LEDGF expression was found in advanced T stage patients (Figure 3D).

Previous studies reported the presence of *SETD2* mutations in A498 cells, resulting in the loss of H3K36me3, which is consistent with our results (Figure 3A). To explore the role of LEDGF in *SETD2* mutant ccRCC, we selected A498 cells for experiments. 786‐O cells, known as H3K36me3^WT^ cells, were also used as the comparison in experiments. We successfully knocked down the expression of LEDGF using siRNA (Figure 3E) and conducted a series of phenotypic experiments. CCK‐8, colony formation and EdU proliferation assays illustrate the same and stable results, that is, knocking down LEDGF could significantly inhibit the proliferation ability of 786‐O cells. The same results were found in A498 cells (Figure 3F–I), further demonstrating that LEDGF is involved in other important regulatory processes besides recognizing H3K36me3. In summary, LEDGF is overexpressed in ccRCC and effectively promotes ccRCC cells proliferation.

### 2.6 CARM1 and H3R17me2a are Overexpressed in ccRCC and Promote Proliferation

**Figure 4:**
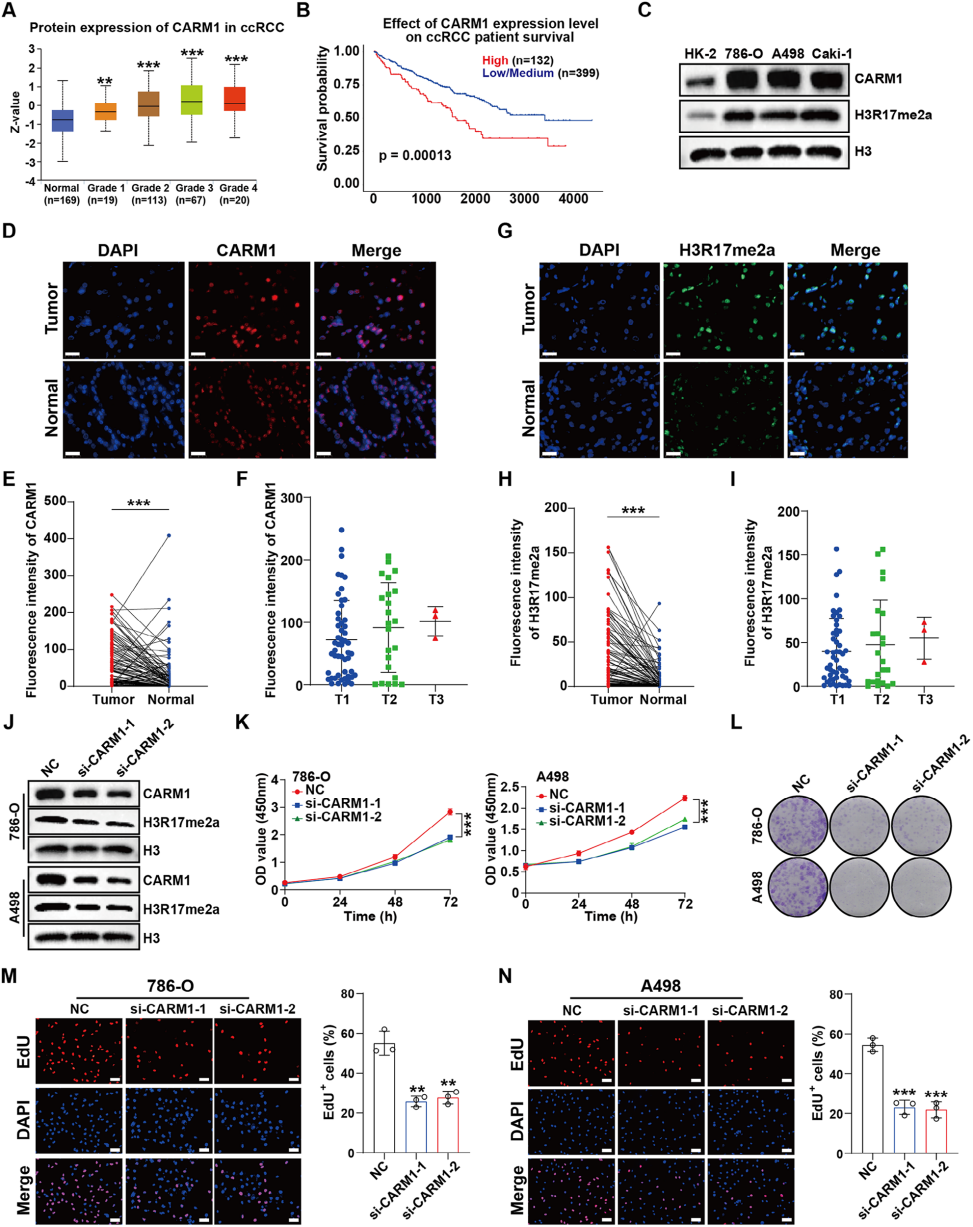
CARM1 is highly expressed in ccRCC and promotes proliferation. A,B) CARM1 is highly expressed in ccRCC patients and is correlated with poor prognosis on the UALCAN database. C) CARM1 and H3R17me2a are highly expressed in ccRCC cells such as 786‐O, A498, and Caki‐1. D,E) Immunofluorescence staining using the tissue microarray revealed that CARM1 is highly expressed in ccRCC tissue samples. Scale bar = 100 µm. (Tumor n = 79, Normal n = 79) F) CcRCC patients with advanced T stage have higher CARM1 expression, which may be associated with poor prognosis. (T1 n = 53, T2 n = 23, T3 n = 3) G,H) Immunofluorescence staining using the tissue microarray revealed that H3R17me2a level is higher in ccRCC tissue samples. Scale bar = 100 µm. (Tumor n = 79, Normal n = 79) I) CcRCC patients with advanced T stage have higher H3R17me2a level, which may be associated with poor prognosis. (T1 n = 53, T2 n = 23, T3 n = 3) J) The siRNA targeting CARM1 mRNA can effectively interfere with CARM1 and decreases H3R17me2a in ccRCC cells. K–N) Reduction of CARM1 and H3R17me2a significantly inhibited the proliferation of ccRCC cells, as demonstrated by CCK‐8 (K), colony formation (L) and EdU proliferation assays (M‐N). Scale bar = 100 µm. Data are shown as mean ± SD. **p < 0.01, ***p < 0.001.

Coactivator‐Associated Arginine Methyltransferase 1 (CARM1) is a protein arginine methyltransferase (PRMT) that acts as a coactivator in a number of transcriptional programs. Previous studies have reported that CARM1 catalyzes H3R17me2a, regulating downstream gene transcription. Results from public databases showed that CARM1 expression was abnormally elevated in ccRCC, especially in the advanced patients (**Figure** **4**A). Meanwhile, patients with high CARM1 expression tended to have worse prognosis (Figure 4B). We evaluated the expression of CARM1 and H3R17me2a levels in ccRCC cell lines. It was found that both CARM1 and H3R17me2a levels were abnormally elevated in ccRCC cells (Figure 4C). The same result was also observed in ccRCC tissues (Figure 4D,E,G,H). Meanwhile, advanced T stage patients had higher expression of CARM1 (Figure 4F) and H3R17me2a level (Figure 4I).

In order to explore the role of CARM1 and H3R17me2a, we first knocked down their expression level using siRNA targeting CARM1 (Figure 4J). We then carried out CCK‐8 (Figure 4K), colony formation (Figure 4L), and EdU proliferation assays (Figure 4M,N). The results showed that CARM1 knockdown led to the reduction of H3R17me2a and significantly inhibited the proliferation of ccRCC cells. These results indicate that there is overexpression of CARM1 in ccRCC, and H3R17me2a may be a cancer‐promoting signal.

### 2.7 LEDGF Interacts with H3R17me2a Cooperatively Promoting ccRCC Cell Proliferation

We constructed plasmids overexpressing CARM1 and PRMT6, respectively, and transfected them into ccRCC cells. Western blot analysis revealed that simultaneous overexpression of CARM1 and PRMT6 significantly increased H3R17me2a in ccRCC cells (Figure S4A,B, Supporting Information). We used LEDGF‐targeting siRNA to treat ccRCC cells with elevated H3R17me2a level and performed a series of phenotype experiments. Results of CCK‐8 (Figure S4C,D, Supporting Information), colony formation (Figure S4E, Supporting Information), and EdU proliferation assays (Figure S4F,G, Supporting Information) illustrated that the reduction of LEDGF can partially compensate for the cancer‐promoting effect of increasing H3R17me2a level.

### 2.8 LEDGF Interacts with H3R17me2a Cooperatively Promoting Proliferation in H3K36me3‐Deficient 786‐O Cells

In order to explore whether the above findings are specific to A498 cells, we intended to knock out SETD2 in 786‐O cells to construct H3K36me3‐deficient cells. Western blot was performed to validate the knockout efficiency of SETD2 and the reduction of H3K36me3 level in pooled 786‐O cells transduced with SETD2‐targeting CRISPR‐Cas9 lentivirus (Figure S5A, Supporting Information). KO‐SETD2#1 786‐O cells were renamed as H3K36me3‐deficient#1 cells and were used in further experiments. We used LEDGF‐targeting siRNA to knock down LEDGF in H3K36me3‐deficient#1 cells (Figure S5B, Supporting Information). CCK‐8 (Figure S5C, Supporting Information), colony formation (Figure S5D, Supporting Information) and EdU proliferation assays (Figure S5E, Supporting Information) were carried out to detect that reduction of LEDGF inhibited proliferation of H3K36me3‐deficient#1 cells. We then reduced CARM1 and H3R17me2a in H3K36me3‐deficient#1 cells using CARM1‐targeting siRNA (Figure S5F, Supporting Information), and carried out CCK‐8 (Figure S5G, Supporting Information), colony formation (Figure S5H, Supporting Information) and EdU proliferation assays (Figure S5I, Supporting Information). The results showed that CARM1 knockdown led to the reduction of H3R17me2a and significantly inhibited the proliferation of H3K36me3‐deficient#1 cells.

We simultaneously overexpressed CARM1 and PRMT6 in H3K36me3‐deficient#1 cells, which can significantly increase H3R17me2a (Figure S5J, Supporting Information). We then used LEDGF‐targeting siRNA to treat the cells with elevated H3R17me2a level and performed CCK‐8 (Figure S5K, Supporting Information), colony formation (Figure S5L, Supporting Information), and EdU proliferation assays (Figure S5M, Supporting Information). The results illustrated that reduction of LEDGF can partially compensate for the cancer‐promoting effect of increasing H3R17me2a level in H3K36me3‐deficient#1 cells, a trend that was concordantly observed in A498 cells.

### 2.9 Construction of LEDGF‐KO and H3R17me2a‐Deficient Cells

**Figure 5:**
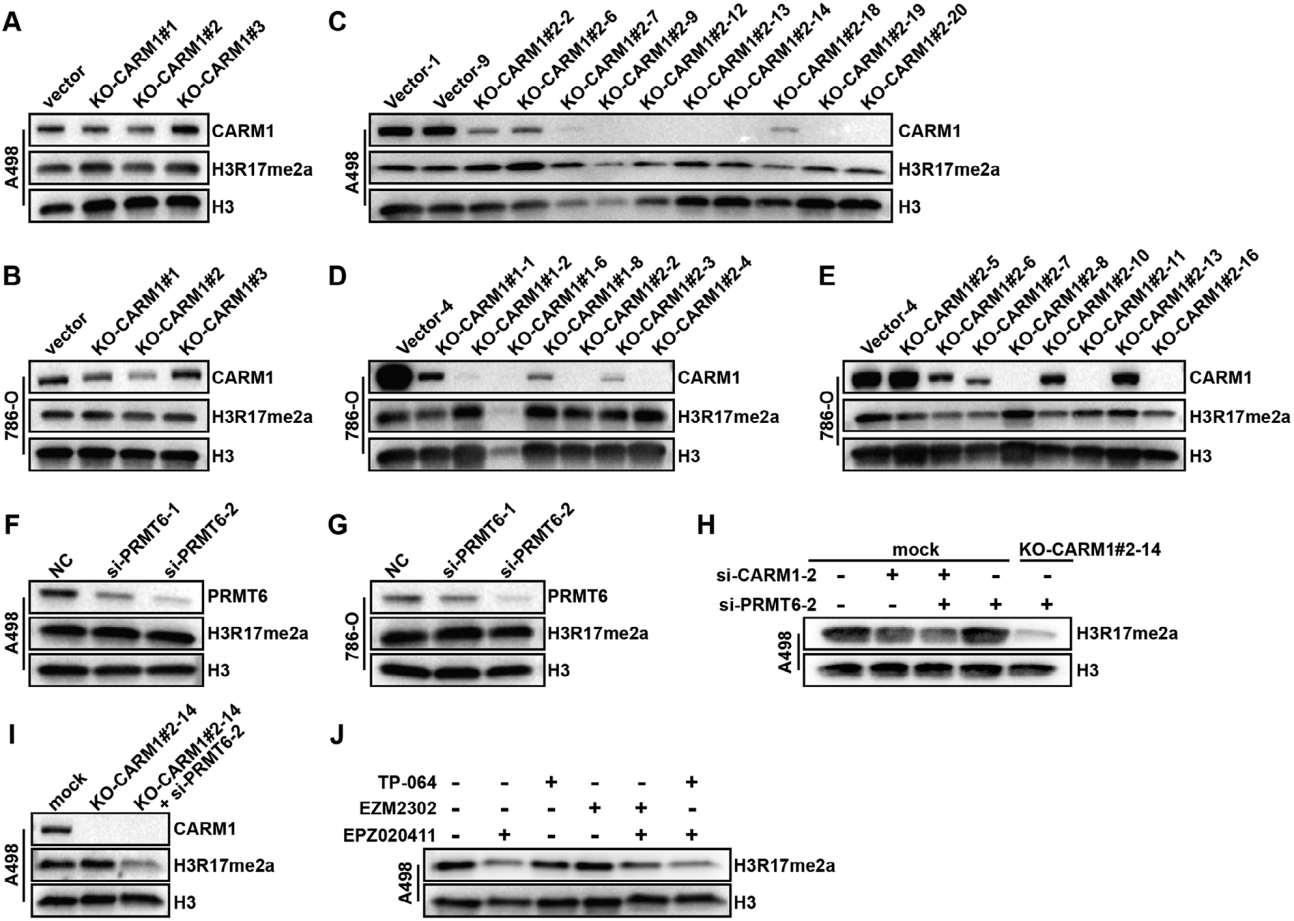
Construction of the H3R17me2a‐deficient cell lines. A,B) Validation of knockout efficiency of CARM1 and the reduction of H3R17me2a level in transduced pooled A498 cells (A) and 786‐O cells (B). C–E) Validation of knockout efficiency of CARM1 and the reduction of H3R17me2a level in monoclonal A498 cells (C) and 786‐O cells (D‐E). F,G) Knockdown of PRMT6 using siRNA has little effect on H3R17me2a level in A498 cells (F) and 786‐O cells (G). H) The combined knockdown of CARM1 and PRMT6 using siRNAs reduces H3R17me2a level in A498 cells; knockdown of PRMT6 in CARM1‐KO cells significantly decreased H3R17me2a in A498 cells. I) Knockdown of PRMT6 in CARM1‐KO cells significantly decreased H3R17me2a in A498 cells. J) The combination of CARM1 (MCE, EZM2302 and TP‐064) and PRMT6 (MCE, EPZ020411) inhibitors can significantly reduce H3R17me2a level in A498 cells.

In order to explore the regulatory mechanism, we constructed the LEDGF‐KO and CARM1‐KO cells by CRISPR‐Cas9‐mediated gene editing. We designed and synthesized the lentiviruses with high infection efficiency (Figures S6A and S7A, Supporting Information). Western blot results showed that the protein levels of LEDGF (Figure S6B, Supporting Information) or CARM1 (**Figure** **5**A,B) were almost eliminated in ccRCC cells after infection with indicated lentivirus. We further used the limiting dilution method for monoclonal cell selection (Figures S6C and S7B, Supporting Information). Western blot results showed that LEDGF protein expression was knocked out in some monoclonal 786‐O cells (Figure S6D,E, Supporting Information) and A498 cells (Figure S6F,G, Supporting Information). Finally, after stable culture, we successfully constructed the LEDGF‐KO ccRCC cell lines (Figure S6H, Supporting Information).

We also used limiting dilution method to generate monoclonal cultures infected with CARM1‐targeting CRISPR‐Cas9 lentivirus and tested the level of CARM1 and H3R17me2a. Interestingly, we found that when CARM1 is deficient in ccRCC cells, a considerable abundance of H3R17me2a can still be detected (Figure 5C–E).

We knocked down PRMT6 in ccRCC cells using siRNA, but there were no significant changes in H3R17me2a level (Figure 5F,G). Subsequently, we interfered with CARM1 or PRMT6 in A498 cells. It was found that transient knockdown of CARM1 with CARM1‐targeting siRNA led to downregulation of H3R17me2a. Simultaneous interference of CARM1 and PRMT6 could lead to further reduction of H3R17me2a (Figure 5H). Interestingly, there was a significant reduction in H3R17me2a level when knocking down PRMT6 in CARM1‐KO cells (Figure 5I). In addition, we selected inhibitors of CARM1 (TP‐064, EZM2302) and PRMT6 (EPZ020411) for further verification. The results showed that the combined inhibition of CARM1 and PRMT6 could effectively reduce the level of H3R17me2a (Figure 5J).

### 2.10 LEDGF Interacts with H3r17me2a Regulating Purine Nucleotide Metabolism

**Figure 6:**
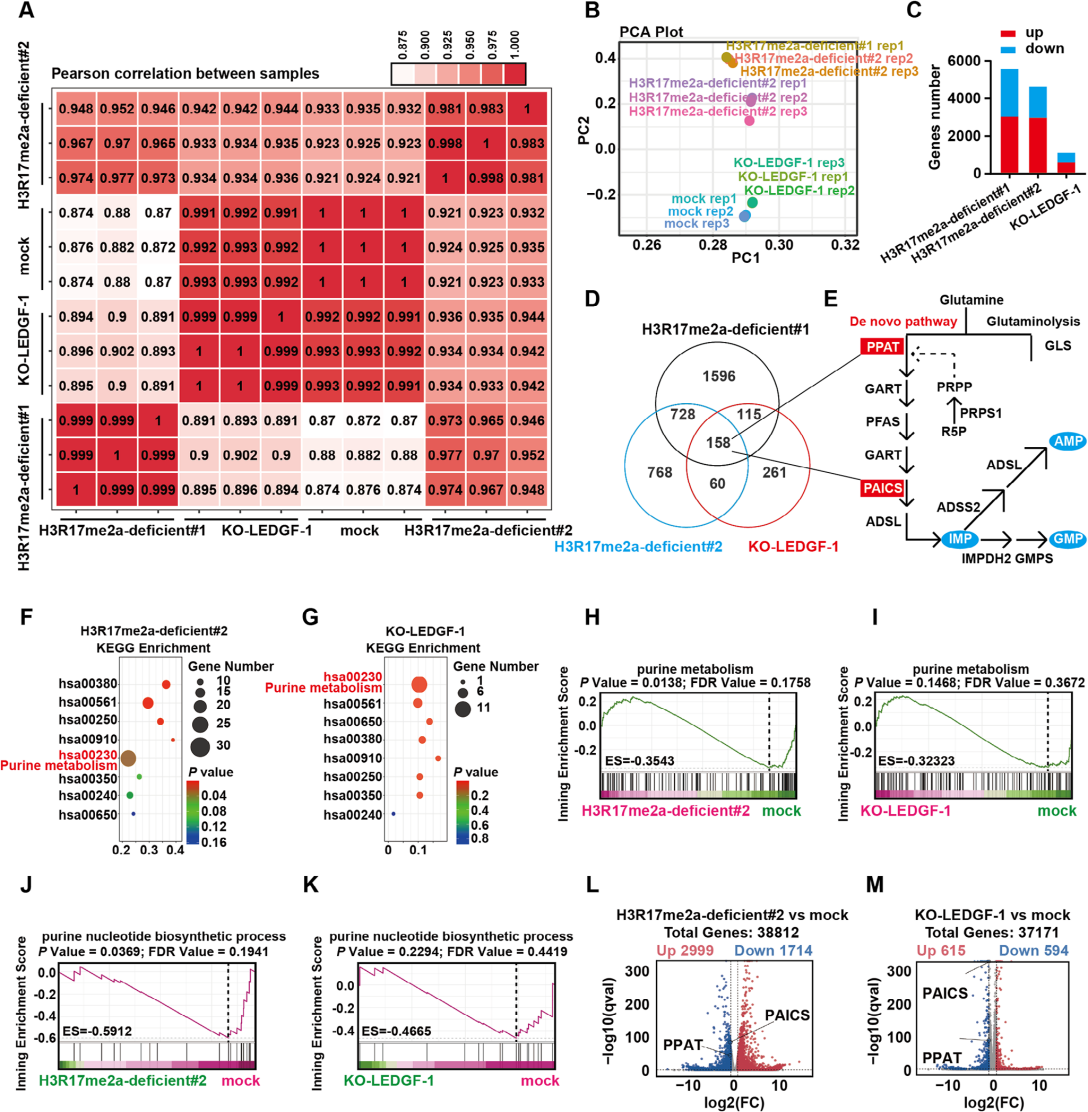
LEDGF reads H3R17me2a regulating purine nucleotide metabolism. A) Heat map shows the Pearson correlation between different samples. B) Principal Component Analysis (PCA) plot shows small intra‐group differences and large inter‐group differences between indicated groups. C) Stack map of the number of differentially expressed genes (DEGs) in different groups. D) Venn diagram shows the intersection of down‐regulated DEGs in each group, including PPAT, PAICS. E) Schematic representation of the de novo synthesis pathway of nucleotides. The blue label indicates the major nucleotide products. F,G) KEGG enrichment analysis of DEGs in different treatment groups shows a close correlation with purine metabolism. H,I) GSEA analysis of purine metabolism in different treatment groups. J,K) GSEA analysis of purine nucleotide biosynthetic process in different treatment groups. L,M) Volcano plots of DEGs in different treatment groups. Key enzymes in the de novo pathway are labeled such as PPAT and PAICS.

To explore target genes regulated by LEDGF and H3R17me2a, we performed RNA sequencing (RNA‐seq) assay on indicated cells. Considering the functional redundancy of PRMT6 and CARM1 in A498 cells, we constructed two cell models with deficient H3R17me2a using combined inhibitors (H3R17me2a‐deficient#1) or knockdown of PRMT6 in CARM1‐KO cells (H3R17me2a‐deficient#2), respectively. Two different H3R17me2a‐deficient cell models were highly correlated (**Figure** **6A,B**).

RNA‐seq results suggested that reduction of LEDGF or H3R17me2a resulted in significant changes in A498 cells (Figure 6C; Figure S8A, Supporting Information). H3R17me2a has been reported to be a marker of gene transcriptional activity, so we focused on the down‐regulated gene sets from the RNA‐seq dataset. Venn diagram showed that 158 genes were significantly down‐regulated in the three groups, including PPAT and PAICS (Figure 6D).

PPAT and PAICS have been reported to be involved in the catabolism of glutamine and are key enzymes in the de novo nucleotide synthesis pathway (Figure 6E). In order to explore the target genes regulated by LEDGF and H3R17me2a, we first performed GSEA analysis of glutamine metabolism. However, the results suggest that changes in LEDGF and H3R17me2a may not be related to glutamine metabolism (Figure S8B–D, Supporting Information). We then performed Kyoto Encyclopedia of Genes and Genomes (KEGG) enrichment analysis on the 3 groups. The results showed that the regulated genes were highly enriched in purine metabolic pathways (Figure 6F,G; Figure S8E, Supporting Information). The subsequent Gene Set Enrichment Analysis (GSEA) obtained consistent results (Figure 6H,I; Figure S8F, Supporting Information). Further, we performed GSEA analysis of purine nucleotide metabolism. The results showed that the expression levels of some purine nucleotide metabolic pathway genes were also reduced when LEDGF knockout or H3R17me2a reduction (Figure 6J,K, Figure S8G, Supporting Information). LEDGF interacts with H3R17me2a and may regulate purine nucleotide metabolism in A498 cells. Finally, the differentially expressed genes are shown in Figure 6L,M and Figure S8H (Supporting Information).

### 2.11 LEDGF Interacts with H3R17me2a Regulating Key Enzymes in the De Novo Nucleotide Synthesis Pathway

**Figure 7:**
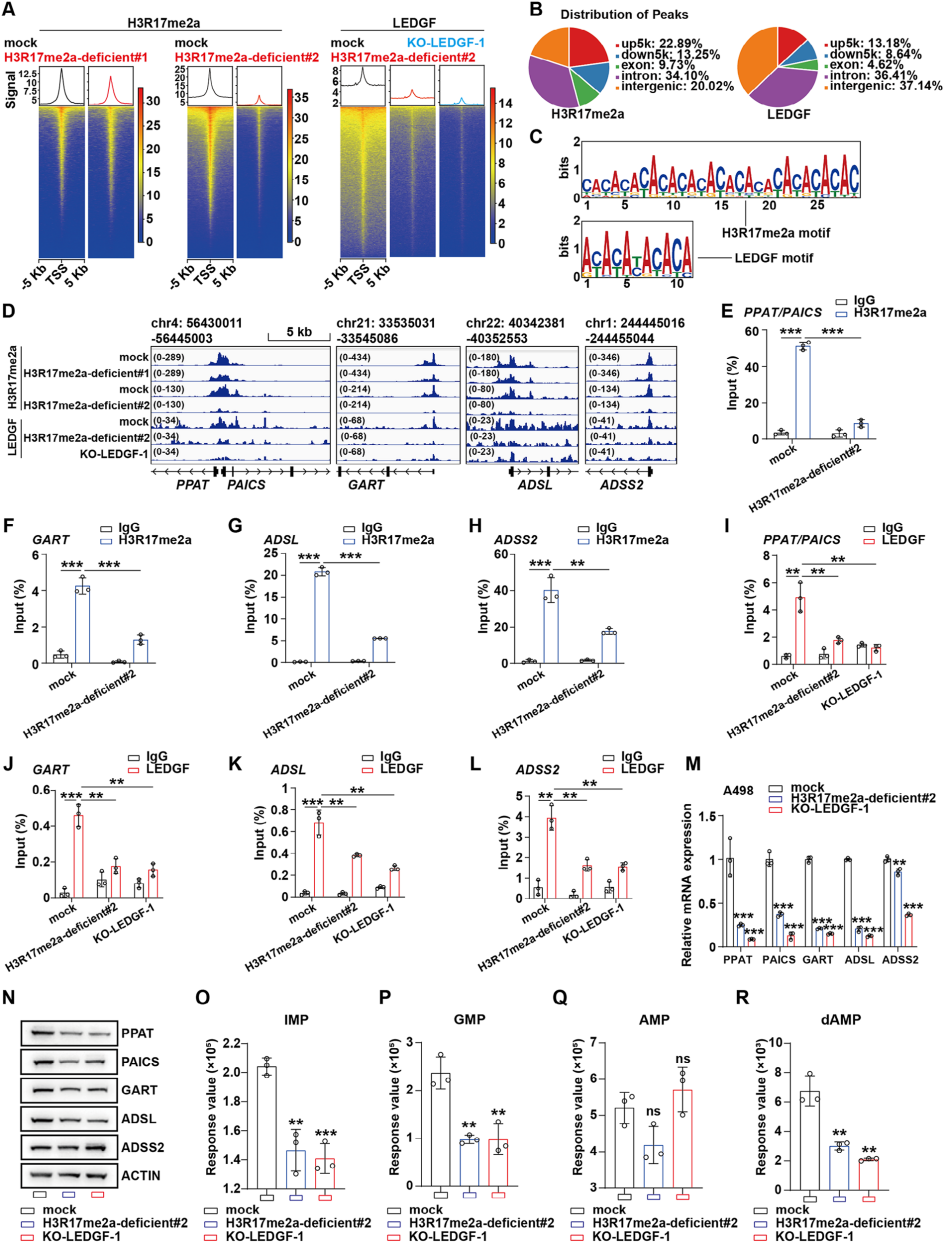
LEDGF reads H3R17me2a regulating key enzymes in the de novo synthesis pathway. A) Heat maps and averaged CUT&Tag signals of H3R17me2a and LEDGF across ±5 kb from the transcription start site (TSS) in A498 cells. B) Distribution of H3R17me2a and LEDGF enrichment peaks on the genome. C) Motif analysis of high frequency enrichment of H3R17me2a and LEDGF shows that there is a high degree of co‐enrichment in the genome. D) There are specific enrichment peaks at the TSS of *PPAT*, *PAICS*, *GART*, *ADSL*, and *ADSS2* in indicated groups, suggesting a potential transcriptional regulatory axis in A498 cells. E–H) The specific enrichment of H3R17me2a at the TSS of *PPAT*, *PAICS* (E), *GART* (F), *ADSL* (G), and *ADSS2* (H) was verified by ChIP‐qPCR assay. I–L) The specific enrichment of LEDGF at the TSS of *PPAT*, *PAICS* (I), *GART* (J), *ADSL* (K), and *ADSS2* (L) was verified by ChIP‐qPCR assay. M,N) QRT‐PCR was used to demonstrate that decrease of LEDGF or H3R17me2a can reduce mRNA expression of key enzymes in the de novo synthesis pathway. (N) Western blot was performed to detect that decrease of LEDGF or H3R17me2a can significantly reduce the protein expression of key enzymes of de novo synthesis, except ADSS2. O–R) Metabolomics results showed that reduction of H3R17me2a level or LEDGF significantly decreases IMP (O) and GMP (P) levels in A498 cells. While the AMP in A498 cells remain relatively stable (Q), there is a significant reduction in dAMP (R) level. Data are shown as mean ± SD. **p < 0.01, ***p < 0.001. ns means no significance.

In order to explore target genes, we performed the Cleavage Under Targets and Tagmentation (CUT&Tag) assay on indicated cells to detect enrichment sites of LEDGF and H3R17me2a on the genome. In both H3R17me2a‐deficient groups, H3R17me2a was less enriched on the genome than in the control group. In particular, H3R17me2a enrichment on the genome was significantly reduced in the H3R17me2a‐deficient#2 group. In addition, LEDGF enrichment on the genome was also significantly reduced in both LEDGF‐KO cells and H3R17me2a‐deficient#2 cells (**Figure** **7**A). We found that LEDGF and H3R17me2a are highly enriched in the up5k and down5k regions of the genome (Figure 7B; Figure S9A, Supporting Information). We carried out motif analysis on the MEME database. The results suggested that the binding motifs of LEDGF and H3R17me2a were highly similar (Figure 7C). KEGG pathway analysis of LEDGF and H3R17me2a enriched genes showed that the two were closely related in tumors, especially in renal cell carcinoma (Figure S9B,C, Supporting Information).

Considering the results of RNA‐seq analysis, we attempted to explore whether LEDGF and H3R17me2a directly regulate transcription of key enzymes in the de novo synthesis pathway. Interestingly, the enrichment peaks of the two are not only found at the transcription start sites (TSS) of *PPAT* and *PAICS*, but also at the TSS of other key genes such as *GART*, *ADSL*, and *ADSS2* (Figure 7D). Subsequently, we conducted ChIP‐qPCR experiments, and the results suggested that both LEDGF and H3R17me2a were enriched at the TSS of the five target genes (*PPAT*, *PAICS*, *GART*, *ADSL*, and *ADSS2*) (Figure 7E–L). We detected the mRNA levels of the five target genes in different groups of A498 cells, and the results showed that reduction of LEDGF or H3R17me2a could significantly down‐regulate the mRNA expression of the target genes (Figure 7M). Western blot results showed that protein levels of PPAT, PAICS, GART, and ADSL were down‐regulated in LEDGF‐KO cells or H3R17me2a‐deficient#2 cells (Figure 7N). However, ADSS2 protein level did not appear to change significantly.

The same detection was repeated in 786‐O cells. Interestingly, we found that the changes in mRNA of target genes were not consistent with the results in A498 cells. For example, reduction of H3R17me2a in 786‐O cells did not lead to down‐regulation of PAICS mRNA level (Figure S9D, Supporting Information). As we suspected, *SETD2* mutations in A498 cells lead to deletion of H3K36me3, which in turn leads to great changes in the epigenetic regulation. We further repeated the experiments with H3K36me3‐deficient#1 cells. Results of qRT‐PCR illustrated that decrease of LEDGF or H3R17me2a level can reduce mRNA expression of key enzymes in the de novo synthesis pathway in H3K36me3‐deficient#1 cells (Figure S9E, Supporting Information), a trend that was concordantly observed in A498 cells.

We then analyzed the mRNA expression correlation between LEDGF and target genes in TCGA‐ccRCC tissues on the GEPIA database. The results suggested that LEDGF was strongly positively correlated with mRNA expression levels of target genes (Figure S10A–E, Supporting Information). We also analyzed the protein expression levels of target genes in ccRCC tissues. The results showed that PPAT, PAICS, GART and ADSL were significantly overexpressed in ccRCC, which was consistent with the expression trend of LEDGF (Figure S10F–I, Supporting Information). As for ADSS2 expression levels, there was no statistical difference between ccRCC tissues and normal tissues (Figure S10J, Supporting Information).

In order to explore the regulation of LEDGF and H3R17me2a on intracellular nucleotide levels, we performed metabolic sequencing for different groups of A498 cells. The results showed that reduction of LEDGF or H3R17me2a significantly reduced intracellular IMP and GMP levels (Figure 7O,P). However, AMP levels in A498 cells did not change significantly (Figure 7Q). We suspect there are several potential reasons. First, the expression level of ADSS2, a key enzyme of AMP metabolism, did not change in indicated groups. Second, the level of intracellular dAMP was significantly reduced (Figure 7R), which may partially maintain the level of AMP.

### 2.12 Potential Positive Feedback may Exist between LEDGF and H3R17me2a

Interestingly, specific enrichment peaks of LEDGF and H3R17me2a in the TSS of *CARM1* were found (Figure S9F, Supporting Information). Results of ChIP‐qPCR also confirm the specific enrichment (Figure S9G,H, Supporting Information). This suggested that there may be positive feedback regulation of CARM1 by LEDGF and H3R17me2a in A498 cells. In fact, significant downregulation of CARM1 mRNA levels did occur after LEDGF knockout (Figure S9I, Supporting Information).

### 2.13 LEDGF Deprivation Significantly Inhibited the Proliferation of Xenograft In Vivo

**Figure 8:**
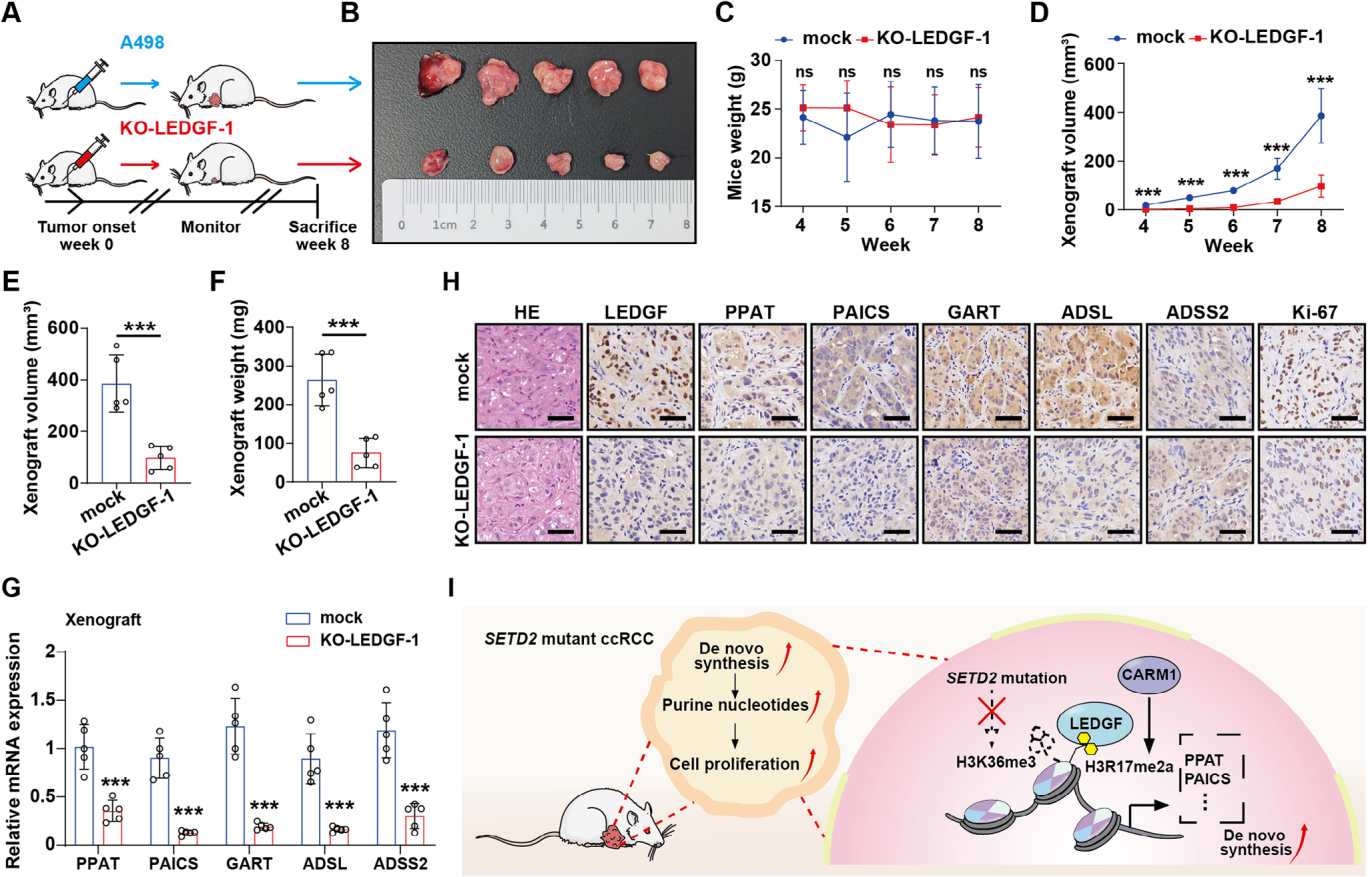
Deficiency of LEDGF protects NKG mice against xenograft proliferation. A) Schematic diagram of subcutaneous tumor model in NKG mice in indicated treatment groups. All surviving mice were euthanized 8 weeks after tumor cell inoculation. B) Knock out of LEDGF effectively reduced the proliferation of xenografts in NKG mice. (n = 5) C) There was no significant difference in body weight between the two groups throughout the experiment. D–F) Elimination of LEDGF effectively reduced the volume (D‐E) and weight (F) of NKG mice xenografts. G) QRT‐PCR was used to demonstrate that decrease of LEDGF can reduce mRNA expression of PPAT, PAICS, GART, ADSL, and ADSS2 in xenograft tumors. H) The proliferation ability of xenografts in LEDGF‐KO group was significantly reduced. The expression levels of PPAT, PAICS, GART, and ADSL were significantly decreased, while ADSS2 expression was almost unchanged. Scale bar = 100 µm. I) A schematic model illustrating that LEDGF interacts with CARM1‐mediated H3R17me2a to promote ccRCC progression. Data are shown as mean ± SD. ***p < 0.001. ns means no significance.

Ten male NKG mice were obtained for this study. After 1 week of adaptation, they were randomly divided into 2 groups with 5 mice in each group. We injected indicated cells in the armpit fat pad of mice. The flow chart is shown in **Figure** **8**A.

Four weeks after the subcutaneous tumor model was established, we observed and recorded the conditions of the two groups once a week. In vivo results suggested that the elimination of LEDGF effectively inhibited the growth of xenografts in mice (Figure 8B). However, there was no significant difference in the body weight between the two groups (Figure 8C). Our previous results showed that the proliferation capacity of LEDGF‐KO A498 cells was significantly reduced, and the same results were also observed in vivo (Figure 8D–F). In addition, qRT‐PCR and immunohistochemical experiments were performed to verify the expression changes of target genes after LEDGF knockout. Both the mRNA and protein expression levels of PPAT, PAICS, GART and ADSL were significantly down‐regulated in the LEDGF‐KO group (Figure 8G,H), while there was no significant change in ADSS2 protein level (Figure 8H), which was consistent with the results of in vitro experiments.

In summary, the present study screened H3R17me2a as a novel LEDGF recognition modification mark. *SETD2* mutant ccRCC is deficient in H3K36me3, and LEDGF interacts with H3R17me2a to regulate the expression of key enzymes in the de novo nucleotide synthesis pathway and facilitate the malignant proliferation of ccRCC (Figure 8I).

## 3 Discussion

In *SETD2* mutant ccRCC, H3K36me3 disappears, and the original LEDGF recognition relationship no longer exists. In the present study, we illustrated for the first time that LEDGF recognizes CARM1‐dependent H3R17me2a. Our results demonstrate that overexpressed LEDGF in ccRCC regulates gene transcription of key enzymes in the nucleotide de novo synthesis pathway by binding to H3R17me2a, leading to metabolic reprogramming of nucleotides, and thereby promoting excessive proliferation of *SETD2* mutant ccRCC cells.

SETD2 can catalyze H3K36me3, which is recognized by LEDGF.^[^ ^24^, ^25^ ^]^ SETD2 has been reported to have high frequency mutations in a variety of tumors, with a mutation rate of 15%–23% in ccRCC patients, which directly leads to a reduction or even deletion of H3K36me3.^[^ ^26^ ^]^ H3K36me3 is a conserved histone methylation marker that mediates several transcription‐related events, such as transcriptional activity regulation, transcription elongation, pre‐mRNA alternative splicing, and RNA m6A methylation.^[^ ^27^ ^]^ In ccRCC, SETD2‐dependent H3K36me3 has been reported to regulate the metastasis process of tumor cells.^[^ ^17^ ^]^ Data of public database shows that *SETD2* has a 13% high frequency mutation in ccRCC and is associated with poor prognosis in patients. However, the mechanism remains unclear. In fact, in *SETD2*‐mutant ccRCC, LEDGF can no longer recognize SETD2‐dependent H3K36me3, and the epigenetic background has been drastically altered, which may be a key factor in the malignant progression of ccRCC. Therefore, this study tried to explore the potential novel transcriptional recognition pattern in *SETD2* mutant ccRCC.

Readers are known as proteins that recognize histone modifications and cause associated biological consequences. Members from a protein family called the Royal Family have PWWP, Tudor, chromo, and MBT domains that recognize methylated histone tails.^[^ ^28^ ^]^ Among them, LEDGF, with a PWWP domain, is reported to be the classic H3K36me3 reader. LEDGF binds to H3K36me3 recruiting RNA polymerase 2 regulating key cellular events. For example, it regulates DNA methylation and promotes DNA mismatch repair.^[^ ^29^ ^]^ In fact, LEDGF, as a chromatin localization protein, plays an important role as the molecular bridge. LEDGF IBD domain can combine the mixed lineage leukaemia (MLL1)‐menin complex4 to regulate Hox gene, and then participate in the occurrence of MLL leukemia.^[^ ^30^ ^]^ In addition, previous studies have focused on exploring the interaction of the LEDGF IBD domain with HIV integrase.^[^ ^31^ ^]^ These studies all highlight the importance of LEDGF, however, the study of LEDGF in cancer is still scarce.

Our previous research found that LEDGF is dysregulated across cancers and is associated with poor prognosis.^[^ ^16^ ^]^ Interestingly, although there was no significant difference in LEDGF mRNA levels in ccRCC compared to normal tissue, its protein levels were significantly up‐regulated. This suggests a potential cancer‐promoting effect of LEDGF in ccRCC. Cell function experiments also showed that LEDGF knockdown could significantly inhibit the proliferation of ccRCC cells. In particular, this phenomenon was observed not only in *SETD2* wild‐type 786‐O cells, but also in *SETD2*‐mutant A498 cells. This result provides strong evidence that LEDGF is involved in a key process in the development of ccRCC and may not be dependent on H3K36me3 deposited by SETD2. In other words, there may be a novel epigenetic pattern based on LEDGF in *SETD2*‐mutant ccRCC. It is highly likely that LEDGF recognizes other histone modification marks mediating new transcriptional axes.

In order to find potential recognition modification marks for LEDGF, we incubated purified LEDGF protein with modified Histone Peptide Array. The results showed that LEDGF had the strongest binding ability to H3R17me2a. Previous studies have reported that the PWWP domain of LEDGF, as a member of the Royal family, recognizes methylation modifications of histone lysine.^[^ ^32^ ^]^ However, it has never been reported that the PWWP domain can recognize methylation modifications of arginine. In the present study, we proved for the first time that LEDGF did indeed bind to H3R17me2a, and the key binding sites were Asn38 and Asp57 of the PWWP domain. This finding not only expands the understanding of the regulatory mechanisms of epigenetic modification, but also fills the gap in the LEDGF‐driven transcriptional reprogramming mechanisms in *SETD2* mutant ccRCC.

CARM1 is a coactivator of many transcription factors. CARM1 and CARM1‐dependent H3R17me2a are associated with transcriptional activation with H3R17me2a recruiting the human RNA polymerase‐associated factor 1 complex.^[^ ^33^ ^]^ Loss of CARM1 in mouse embryos leads to loss of estrogen response and reduced expression of some estrogen receptor‐α (ERα) target genes. A recent study used a protein domain microarray approach to identify an effector protein, TDRD3, as a “reader” for H3R17me2a.^[^ ^34^ ^]^ TDRD3 contains a typical Tudor domain that mediates methyl‐specific binding. In addition, TDRD3 acts as a coactivator in estrogen response elements (ERE) ‐luciferase reporter assays, while endogenous TDRD3 can be detected on pS2 promoter in ChIP assays.^[^ ^34^ ^]^ Similarly, LEDGF PWWP, as a member of the Tudor domain, can also recognize H3R17me2a and mediate transcriptional regulation in the present study.

When constructing the CARM1‐KO cell lines, we noticed something interesting. After knockdown of CARM1 using siRNA, H3R17me2a levels in ccRCC cells were significantly down‐regulated. However, in monoclonal CARM1‐KO cell lines, H3R17me2a levels were not significantly down‐regulated, even though CARM1 protein had tended to be unexpressed. It seems that there may be another enzyme that compensates for the changes in H3R17me2a levels. Similarly, Cheng et al found that H3R17me2a levels did not decrease significantly in CARM1‐KO mice. They screened for type I PRMTs and found that PRMT6 can also deposit H3R17me2a in vitro. In addition, they found that the combination of inhibitors of CARM1 and PRMT6 effectively inhibited the proliferation of mouse embryonic fibroblasts, indicating a synergistic effect between CARM1 and PRMT6 inhibition.^[^ ^35^ ^]^ Their results provide direct evidence that PRMT6 also partially catalyzes H3R17me2a and acts redundantly with CARM1. However, this phenomenon has not been reported in ccRCC cells. Therefore, we consider whether there is functional redundancy of PRMT6 and CARM1 in ccRCC. When PRMT6 was knocked down alone, H3R17me2a levels did not change significantly. However, the combination of PRMT6 and CARM1 siRNAs or inhibitors significantly down‐regulated H3R17me2a. Particularly, knocking down of PRMT6 in CARM1‐KO cells significantly down‐regulated the expression of H3R17me2a. The results demonstrated that functional redundancy of PRMT6 and CARM1 also exists in ccRCC.

Through RNA‐seq and CUT&Tag experiments, we found that LEDGF's recognition of H3R17me2a may regulate the expression of key enzymes in the nucleotide de novo synthesis pathway such as PPAT and PAICS. ChIP‐qPCR results showed that both LEDGF and H3R17me2a are highly enriched in the promoter of these target genes, suggesting a potential transcriptional regulatory relationship. Metabolomics sequencing results showed that IMP and GMP were significantly reduced in LEDGF‐KO and H3R17me2a‐deficient cells, further demonstrating LEDGF‐mediated nucleotide reprogramming. Purine nucleotides are essential for RNA and DNA synthesis, signal transduction, metabolism, and energy homeostasis. To synthesize purine nucleotides, cells use the de novo pathway and the salvage pathway. It is traditionally believed that malignant proliferation of tumor cells mainly depends on de novo synthesis pathway.^[^ ^36^ ^]^ ccRCC is characterized by abnormal proliferation of cancer cells, and in order to meet the rapid proliferation of cells, pathways such as glucose and nucleotide metabolism are often reprogrammed. The present study is the first to identify LEDGF‐mediated nucleotide reprogramming in ccRCC. Down‐regulation of LEDGF or H3R17me2a can reduce the nucleotide level in *SETD2* mutant ccRCC. It is worth noting that some of the results in 786‐O and A498 do not match. For example, we found that knocking down LEDGF or H3R17me2a changed ADSS2 protein levels in 786‐O, but not in A498. This suggests that *SETD2* mutation may be associated with unknown changes. In addition, we found that LEDGF and H3R17me2a are highly enriched at the promoter of *CARM1*. QPCR results also suggested a possible positive feedback regulatory relationship between CARM1 and H3R17me2a through LEDGF.

The present study still has limitations. Our results identified a novel transcriptional recognition pattern that H3R17me2a recognized by LEDGF in *SETD2* mutant ccRCC. However, we did not delve into whether this transcription recognition pattern still plays an important role in *SETD2* wild type ccRCC. *SETD2* wild type ccRCC cells and *SETD2* mutant ccRCC cells had certain genetic background differences, and more experiments must be performed for indispensable verification in future. Another potential limitation of this study lies in the relatively small sample size employed of the in vivo experiments. Future investigations should consider incorporating expanded cohort sizes and additional experimental groups to enhance the statistical power and further validate our conclusions.

In summary, we screened H3R17me2a as a novel LEDGF recognition modification mark. In *SETD2* mutant ccRCC, LEDGF recognized H3R17me2a and activated the transcription of key enzymes in the nucleotide de novo synthesis pathway such as *PPAT*, promoting the proliferation of ccRCC. This novel identification helped design novel treatment strategies for *SETD2* mutant ccRCC patients.

## 4 Experimental Section

### Ethical Statement and Sample Acquirement

The ccRCC tissue microarray used in this study was purchased from Hunan Aifang Biotechnology Co., Ltd (Changsha, China). The Ethics Committee of Wuxi No.2 People's Hospital affiliated with Jiangnan University authorized this project (2023‐Y‐166). The animal experiment in this study was approved by the Experimental Animal Management and Animal Welfare Ethics Committee of Jiangnan University (JN. No20240915b0181115).

### Online Databases

Information was retrieved on 830 ccRCC samples from five datasets in the cBioPortal database (https://www.cbioportal.org/).^[^ ^17^ ^]^ The mutation information of *SETD2* was analyzed in ccRCC, and further conducted survival analyses on the cBioPortal database.

The features for the domains and regions of LEDGF were obtained from the UniProt database (https://www.uniprot.org/).^[^ ^37^ ^]^ GeneMANIA database (http://genemania.org/)^[^ ^38^ ^]^ and STRING database (https://string‐db.org/)^[^ ^39^ ^]^ were used to search for proteins that might bind to LEDGF. Further scores were predicted in disease based on the LEDGF protein‐protein interaction network via the canSAR database (https://cansar.ai/).^[^ ^40^ ^]^ Expression of mRNA and protein was detected on the GEPIA database (http://gepia2.cancer‐pku.cn/)^[^ ^41^ ^]^ and UALCAN database (https://ualcan.path.uab.edu/),^[^ ^42^ ^]^ respectively. In addition, the correlation of gene expression was calculated using the GEPIA database. All online databases searched in this study were accessed on March 2025.

### Cell Culture

Human ccRCC cell lines (i.e., 786‐O, A498, Caki‐1) and human proximal tubular cell line (i.e., HK‐2) were acquired from Wuhan Pricella Biotechnology Co., Ltd (Wuhan, China) in May 2023. All cell lines used in this study were tested and authenticated by DNA sequencing using the STR method, and mycoplasma contamination was excluded. The appropriate culture medium was selected for different cells according to Pricella Biotechnology's cell culture manual, and 10% fetal bovine serum (Meisen CTCC, Zhejiang, China) with 1% penicillin/streptomycin (Gibco, America) were used to culture all cell lines at 37 °C in a 5% CO_2_ atmosphere.

### Purified LEDGF Protein and Modified Histone Peptide Array

Purified LEDGF protein was obtained from General Biosystems (Anhui) Co., Ltd (Anhui, China). Briefly, the process involved first constructing the LEDGF expression plasmid and then transfecting it into *E. coli*. The transfected *E. coli* was amplified, followed by protein extraction and purification, ultimately resulting in obtaining high‐purity LEDGF protein.

LEDGF protein was incubated with the modified Histone Peptide Array (Active Motif, 13 005) according to the manufacturer. The modified Histone Peptide Array is a valuable research tool that can be used to screen antibodies, enzymes, and proteins for cross‐reactivity or binding interactions with histones and their post‐translational modifications (PTM). The array screen 59 acetylation, methylation, phosphorylation, and citrullination modifications on the N‐terminal tails of histones H2A, H2B, H3, and H4. Each peptide array contains 384 unique histone modification combinations in duplicate, including up to four separate modifications on the same 19mer peptide, offering the most extensive coverage available for commercial arrays of similar format. This extensive coverage of histone modifications enables the study of not only individual sites, but also the effects of neighboring modifications on recognition and binding. The unique synthesis and conjugation methods ensure greater than 95% purity of bound peptides and high peptide density at each spot, which was advantageous for analysis of interaction sites with low binding constants.

At room temperature (RT), after blocking in 5% milk in TBST (150 mm NaCl, 2 mm KCl, and 25 mm Tris pH 7.4) for 2 h, the arrays were incubated with purified LEDGF protein in binding buffer (TBST, 1% milk, 0.05% NP‐40, 0.1 mm DTT, 0.1 mm PMSF, and protease inhibitor cocktail (MedChemExpress, HY‐K0010)) at 4 °C overnight. After washing three times with TBST, the arrays were soaked in the anti‐LEDGF primary antibody (Abcam, ab177159) at RT for 3 h. Following another three washes with TBST, the arrays were incubated with the corresponding secondary antibody (Proteintech, SA00001‐2) at RT for 2 h. Finally, after washing three times with TBST, enhanced chemiluminescence (Tanon, China) was used to detect the result.

### Molecular Docking Analysis

The molecular docking analysis was performed using the Glide module (Schrödinger, version 2019‐3) to characterize the interaction between LEDGF and the H3R17me2a. The structure of LEDGF PWWP domain (PDB ID 2M16) was obtained from the Protein Data Bank (PDB) database (https://www.rcsb.org/). The structure of H3R17me1 (PDB ID 5DWQ) was also derived from the PDB database, based on which an additional methyl group was manually added to lysine 17 to get the H3R17me2a. To prepare the protein, hydrogen atoms were added, water molecules and ions were removed, and the protein's structure was minimized using OPLS3e. The Vander Waals radii were adjusted to 0.8 and a partial charge cutoff of 0.15 was implemented for the docking parameters. The docking task utilized standard‐precision peptide without specifying LEDGF PWWP domain active site residues. The docking results were visualized using PyMol (version 2.4).

### CRISPR‐Cas9 Lentivirus Production, Monoclonal Cell Lines, siRNA, and Plasmids Transfection

The LEDGF‐targeting and CARM1‐targeting CRISPR‐Cas9 lentivirus were designed and synthesized by Shanghai Genechem Co.,Ltd (Shanghai, China). The sequences of sgRNAs were as follows: LEDGF‐sgRNA‐1: 5′‐GGTGGCTTTACAGCTCCATC‐3′, LEDGF‐sgRNA‐2: 5′‐AGATGAAAGGTTATCCCCAT‐3′, LEDGF‐sgRNA‐3: 5′‐CTTTCATCTTGGCGAAGATG‐3′; CARM1‐sgRNA‐1: 5′‐AGAGACAGAGTGCAGCCGTG‐3′, CARM1‐sgRNA‐2: 5′‐TGTGTTCAGCGAGCGGACGG‐3′, CARM1‐sgRNA‐3: 5′‐ATGCAGGACTACGTGCGGAC‐3′. Lentivirus infection was performed according to the manufacturer. Monoclonal cell lines were established using the limiting dilution method. Briefly, qualified pooled cells were selected by western blot. Cells were seeded into 96‐well plates at a density of 0.5 cells per well. At day 3 of culture, wells containing single clones were identified and marked. On day 14, cellular proliferation in the selected wells was photographically documented. Ultimately, appropriately monoclonal cell lines were chosen for subsequent experiments.

The SETD2‐targeting CRISPR‐Cas9 lentivirus was designed and synthesized by Beijing Qingke Biotechnology Co., Ltd (Beijing, China). The sequences of sgRNAs were as follows: SETD2‐sgRNA‐1: 5′‐TCACTCGAGCTTTAAACTCT‐3′, SETD2‐sgRNA‐2: 5′‐CAGTTGTCCGTTCACAGTCC‐3′, SETD2‐sgRNA‐3: 5′‐TGAGTTCGATCATACACAAC‐3′, SETD2‐sgRNA‐4: 5′‐CTTATCGAGAGAGGACGCGC‐3′. Lentivirus infection of pooled 786‐O cells was performed according to the manufacturer.

The siRNAs used in this study were obtained by Beijing Tsingke Biotech Co., Ltd. The plasmids used in this study were purchased from Wuhan Genecreate Biological Engineering CO., LTD (Wuhan, China). All plasmids were confirmed by Sanger sequencing before use. Lipo3000 was used for siRNAs and plasmids transfection. The sequences of siRNAs were as follows: LEDGF siRNA‐1: 5′‐GGAAGAUACCGACCAUGAA‐3′, LEDGF siRNA‐2: 5′‐GCAGCAACUAAACAAUCAA‐3′, LEDGF siRNA‐3: 5′‐AGACGAAGUUCCUGAUGGA‐3′; CARM1 siRNA‐1: 5′‐GUCUGCUUAUUGCCAACAA‐3′, CARM1 siRNA‐2: 5′‐GCUACAUGCUCUUCAACGA‐3′, CARM1 siRNA‐3: 5′‐CGACCAACACCAUGCACUA‐3′; PRMT6 siRNA‐1: 5′‐GGCAUUCUGAGCAUCUUCU‐3′, PRMT6 siRNA‐2: 5′‐ACCAGUGGAGACUGUAGAGUU‐3′, PRMT6 siRNA‐3: 5′‐GCAAGACACGGACGUUUCA‐3′.

### Cell Proliferation Assays

Cells (3 × 10^3^) were seeded into 96‐well plates. At each time point, 10 µl of CCK‐8 reagent (Yeasen, China) was mixed with the medium and incubated the cells at 37 °C for 1 h. Finally, optical density (OD) value was measured at 450 nm. In the colony formation experiment, 1000/well cells were cultured at 37 °C and 5% carbon dioxide for 10 days. Finally, the cells were fixed and stained with methanol and crystal violet, respectively. The EdU Cell Proliferation Assay kit (Beyotime, China) was used to detect the proliferation ability of ccRCC cells, which was strictly operated according to the manufacturer. Three independent experiments were performed.

### RNA Isolation and Quantitative Real‐Time PCR (qRT‐PCR)

Trizol Regent (Invitrogen, America) was utilized to isolate total RNA. HiScript III SuperMix (Vazyme, China) was used to perform the reverse transcription for mRNAs. QRT‐PCR was used to analyze the mRNA expression, which was performed by SYBR Green Kit (Yeasen, China) using LightCycler 96 SW 1.1 system (Roche, Switzerland). The value of comparative cycle threshold was analyzed to determine expression. The primer sequences were as follows: PPAT‐F: 5′‐GATGGGAGTTCGGTGCCAA‐3′, PPAT‐R: 5′‐CAACGAAGGGCTGACAATTTTC‐3′, PAICS‐F: 5′‐TTGCAGAAGAATAGCAACTGGTT‐3′, PAICS‐R: 5′‐CACTGTGGGTCATTATTGGCAT‐3′, GART‐F: 5′‐GGAATCCCAACCGCACAATG‐3′, GART‐R: 5′‐AGCAGGGAAGTCTGCACTCA‐3′, ADSL‐F: 5′‐GCTGGAGGCGATCATGGTTC‐3′, ADSL‐R: 5′‐TGATAGGCAAACCCAATGTCTG‐3′, ADSS2‐F: 5′‐TGGTGCCTTTCCTACAGAGC‐3′, ADSS2‐R: 5′‐TGAGCAAAACGAGGTCCAACC‐3′, CARM1‐F: 5′‐CAGCACCTACAACCTCAGCA‐3′, CARM1‐R: 5′‐GGCTGTTGACTGCATAGTGG‐3′.

### Protein Extraction and Western Blot

RIPA (Beyotime, China) mixed with a protease inhibitor (Beyotime) was used to extract cell protein. After incubating RIPA with cells on ice for 10 min, cells were collected into the centrifuge tube using a clean cell scraper. Subsequently, the cells were subjected to ultrasonic crushing treatment, so that the cell membrane and nucleus were broken. The protein supernatant was then collected by centrifugation. BCA protein concentration determination kit (Beyotime) was used to quantify the protein concentration level.

The proteins were separated by SDS‐page electrophoresis, then transferred to PVDF membranes and soaked in 10% milk at RT for 2 h. Subsequently, the membrane was soaked in the primary antibody and incubated at 4 °C for 12 h. After washes of TBST for three times, the membrane was soaked at RT with the corresponding secondary antibody for 2 h. Finally, enhanced chemiluminescence was used to detect the blot. The antibodies used in this study are as follows: LEDGF (Abcam, ab177159), CARM1 (CST, #3379), H3R17me2a (Acive motif, #39 710), PRMT6 (Abcam, ab271091), H3K36me3 (CST, #4909), PPAT (Proteintech, #15401‐1‐AP), PAICS (Proteintech, #12967‐1‐AP), GART (Proteintech, #13659‐1‐AP), ADSL (Proteintech, #15264‐1‐AP), ADSS2 (Proteintech, #16373‐1‐AP), Histone H3 (Proteintech, #17168‐1‐AP), Alpha Actin (Proteintech, #23660‐1‐AP), Flag (Abmart, #M20008), Goat Anti‐Rabbit IgG (H + L) (Proteintech,#SA00001‐2), and Goat Anti‐Mouse IgG (H + L) (Proteintech, #SA00001‐1).

### Immunofluorescence

The cells were fixed in 4% paraformaldehyde at RT for 15 min, and then incubated in PBS containing 0.25% Triton X‐100 for 10 min to make the cells permeable. The fixed cells were first stained with the primary antibody and then stained with corresponding secondary antibody. The nucleus was stained with DAPI. The fluorescence signal was detected using a confocal microscope (FV1000, Olympus).

The PANNORAMIC panoramic slicing scanner (3DHISTECH, Hungary) was utilized to digitize tissue slices and upload them to the computer. The slices were progressively scanned under the scanner's lens, generating a folder that contains comprehensive tissue information. Once this folder was opened using CaseViewer 2.4 software (3DHISTECH, Hungary), the images could be observed with arbitrary magnification ranging from 1× to 400×. The TMA (Tissue Microarray) plug‐in in the Halo v3.0.311.314 analysis software (Indica labs, America) was employed to define the chip tissue point diameter, as well as the number of rows and columns, after which the software automatically assigns unique identifiers. Using the Quantification FL v2.1 module within the Halo v3.0.311.314 analysis software, the positive area, total tissue area, and positive intensity of the target region were quantified.

### Biotinylated Peptide Pull‐Down Assays

The biotinylated histone peptides used in the study were obtained by Nanjing GenScript Biotechnology Co., Ltd (Nanjing, China). The sequences of peptides used in this study were as follows: Histone H3: ARKSTGGKAPRKQLATKAARK(biotin), H3R17me2a: ARKSTGGKAPR(me2a)KQLATKAARK(biotin), H3K36me3: TKVARKSAPATGGVK(me3)KPHRYK(biotin). The purity of all peptides was above 90%. 20 µg of peptide was mixed with the cell lysate, supplemented with PBS to 1000 µl, and then incubated at 4 °C overnight. The strep‐beads (Thermo 20 359 Agarose Resin, America) were added to the mixture and were incubated at 4 °C for 2 h. Subsequently, the beads were washed according to the manufacture. Finally, western blot was performed to detect the result.

### Co‐Immunoprecipitation Assay (Co‐IP)

The total protein samples were prepared with RIPA buffer and then subjected to brief ultrasonic treatment and high‐speed centrifugation. The extracted samples were incubated with the primary antibody (LEDGF, Abcam, ab177159) overnight on a rotary at 4 °C. The protein A magnetic beads (MCE, China) were added and incubated at 4 °C for 3 h. After wash with PBST (PBS + 0.5% Tween‐20, pH 7.4) for 3 times, the magnetic beads were suspended in 1×loading buffer, boiled at 99 °C for 5 min, and then western blot was performed to detect the result. Enhanced chemiluminescence was ultimately used to detect the blot.

### RNA Sequencing (RNA‐Seq) and Data Analysis

Total RNA was extracted from cells by Trizol Regent and RNA sequencing was performed by Hangzhou Lianchuan Biological Information Co.,Ltd (Hangzhou, China). Bioinformatics analyses were carried out on the OmicStudio platform (https://www.omicstudio.cn/home).

### Cleavage Under Targets and Tagmentation (CUT&Tag) Assays

TD904, a Hyperactive Universal CUT&Tag Assay Kit for Illumina Pro, was used in the CUT&Tag experiment. It was obtained from Nanjing Vazyme Biotech Co., Ltd (Nanjing, China). In strict accordance with the instructions, the CUT&Tag experiments were carried out and sent the product to Hangzhou Lianchuan Biological Information Co., Ltd for deep sequencing. Correlation analysis was done in Vazyme cloud platform (http://cloud.vazyme.com:83/).

### Chromatin Immunoprecipitation (ChIP)

The ChIP assays were carried out with A498 cells using Cell Signaling Technology ChIP kit (CST, America). The rabbit IgG was served as the control. ChIP samples were analyzed by qRT‐PCR using the SYBR Green PCR Mix (Q712, Vazyme, China). The primer sequences for ChIP assay were as follows: PPAT/PAICS‐F: 5′‐CAGCTCAGCCCTGCTCCTC‐3′, PPAT/PAICS‐R: 5′‐CGCTACTGTTGGGTGCTGGAAA‐3′, ADSL‐F: 5′‐AGCTCCAGAAGAGTTGAAGCAA‐3′, ADSL‐R: 5′‐GTGATAAGGGGAGGGAGGGAAT‐3′, ADSS2‐F: 5′‐GCCACCCTCCAGCCAGTC‐3′, ADSS2‐R: 5′‐GCCACCAGGGCCTCTTCC‐3′, GART‐F: 5′‐GCCTTCCTGATTGGCTCCAAA‐3′, GART‐R: 5′‐AGAGACGGCTCCTGTAATGG‐3′, CARM1‐F: 5′‐TGAGGGCTGCCTCACAAA‐3′, CARM1‐R: 5′‐CTCATTAACGGCTGCTTTCG‐3′.

### Subcutaneous Tumorigenesis Model

Six‐week‐old male NKG mice (NOD‐*Prkdc^scid^Il2rg^em1/Cyagen^*) were purchased from Saiye(Suzhou) Biological Information Technology Co., Ltd (Suzhou, China). Every 3 × 10^6^ cells were suspended in a mixture of 100 µL PBS and Matrigel (Corning, America) and then injected subcutaneously into the right axilla of NKG mice. The length and width of the xenografts were measured weekly. The tumor volume was calculated as 0.5× length×width^2^. All surviving mice were euthanized 8 weeks after tumor cell inoculation, and related tests were performed. The xenografts from the mice were collected for further analysis.

### Immunohistochemical (IHC) Staining

IHC assay was performed on the paraffin‐embedded (FFPE) xenograft tumors. The sections were incubated with primary antibodies, including anti‐LEDGF (1:100 dilution; Abcam, ab177159), anti‐PPAT (1:100 dilution; Proteintech, #15401‐1‐AP), anti‐PAICS (1:100 dilution; Proteintech, #12967‐1‐AP), anti‐GART (1:100 dilution; Proteintech, #13659‐1‐AP), anti‐ADSL (1:100 dilution; Proteintech, #15264‐1‐AP), anti‐ADSS2 (1:100 dilution; Proteintech, #16373‐1‐AP), and anti‐Ki‐67 (1:4000 dilution; Proteintech, #27309‐1‐AP) followed by incubation with horseradish peroxidase‐conjugated goat anti‐rabbit secondary antibodies for 1 h at RT. Antibody binding was visualized using a 2‐Solution DAB Kit (Invitrogen).

### Statistical Analysis

Data were presented as the mean ± SD. Paired or nonpaired 2‐tailed t‐test was used for comparisons between the two groups. All statistical analyses and visualizations were performed using GraphPad Prism (version 8.0.2). The sample size of each experimental group is shown in each figure as the number of dots. *P* < 0.05 was considered significant. Statistical significance levels were denoted as follows: *p < 0.05, **p < 0.01, and ***p < 0.001.

### Ethics Approval Statement

The Ethics Committee of Wuxi No.2 People's Hospital affiliated with Jiangnan University authorized this project (2023‐Y‐166). The animal experiment in this study was approved by the Experimental Animal Management and Animal Welfare Ethics Committee of Jiangnan University (JN.No20240915b0181115).

## Conflict of Interest

The authors declare no conflict of interest.

## Supporting information



Supporting Information

## Data Availability

The data that support the findings of this study are available from the corresponding author upon reasonable request.
