# Supplementary material for: LEDGF Binds H3R17me2a Promoting De Novo Nucleotide Biosynthesis in SETD2 Mutant Clear Cell Renal Cell Carcinoma
Source: Adv Sci (Weinh). 2025 Jun 23;12(35):e16809. doi: 10.1002/advs.202416809 (PMC12463125; doi:10.1002/advs.202416809)
Supplement: Supplementary file 1 — Supporting Information [file ADVS-12-e16809-s001.docx]

Supporting Information

**LEDGF Binds H3R17me2a Promoting De Novo Nucleotide Biosynthesis in *SETD2* mutant Clear Cell Renal Cell Carcinoma**

*Yuwei Zhang, Yuhua Zhou, Yuezhou Zhang, Jing Lv, Yang Shen, Dong Zhang, Bo liu, Wei Zhao***, Junyi Ju***, Qingyi Zhu*, Ke Wang***, Ninghan Feng**

**
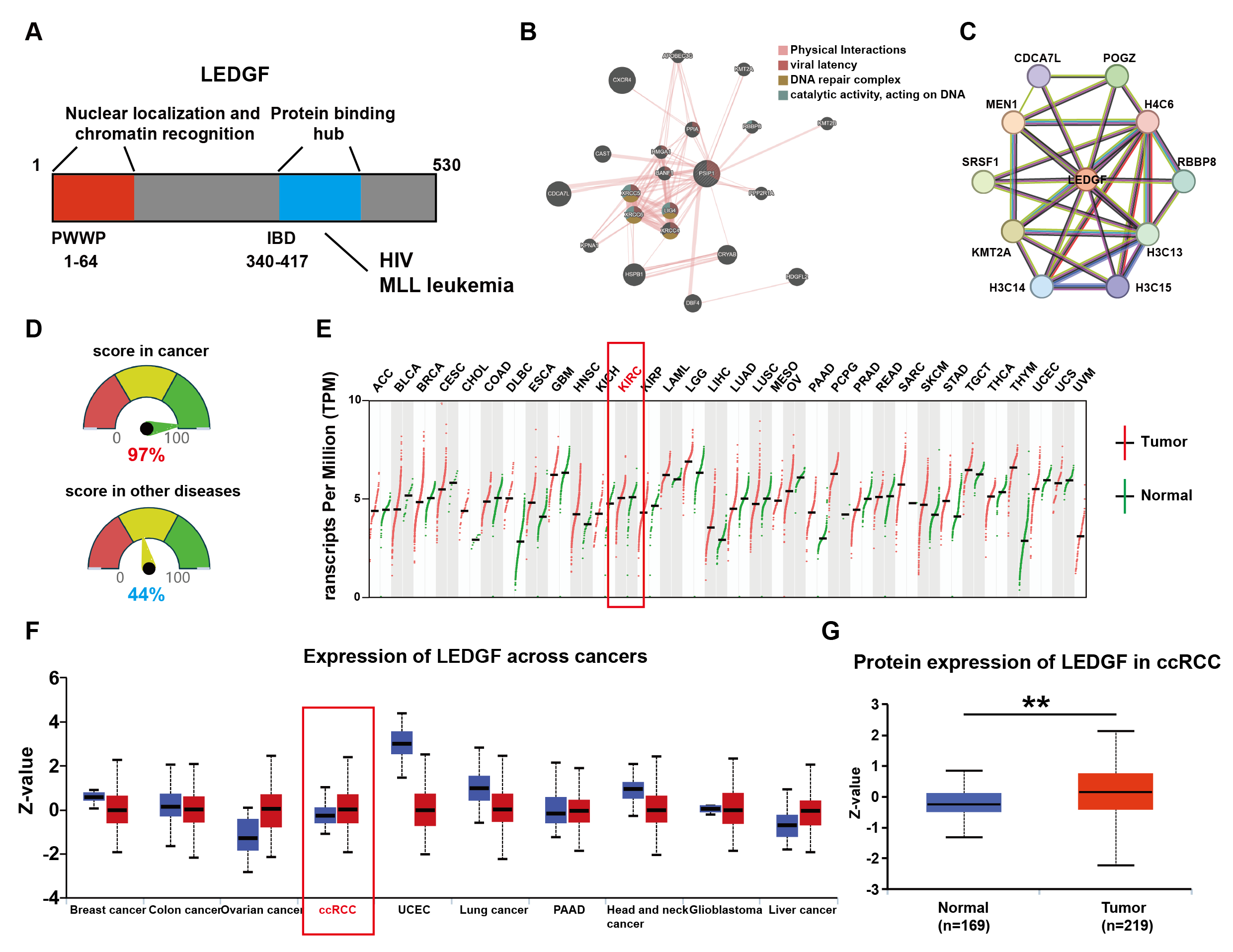
**

**Figure S1. LEDGF is cancer-related and is highly expressed in ccRCC.**

(A) The schematic diagram shows the PWWP and IBD domains of LEDGF protein from the UniProt database (ID O75475). (B-C) The PPI network demonstrates the potential of LEDGF to bind to chromosomes and histones from the GeneMANIA database (B) and STRING database (C). (D) LEDGF is associated with disease, particularly cancer from the canSAR database. (E) The mRNA expression of LEDGF is different across cancers based on the GEPIA database. (F) The protein expression of LEDGF is different across cancers based on the UALCAN database. (G) LEDGF is highly expressed in ccRCC based on the UALCAN database. Data are shown as mean ± SD. ***P* < 0.01.


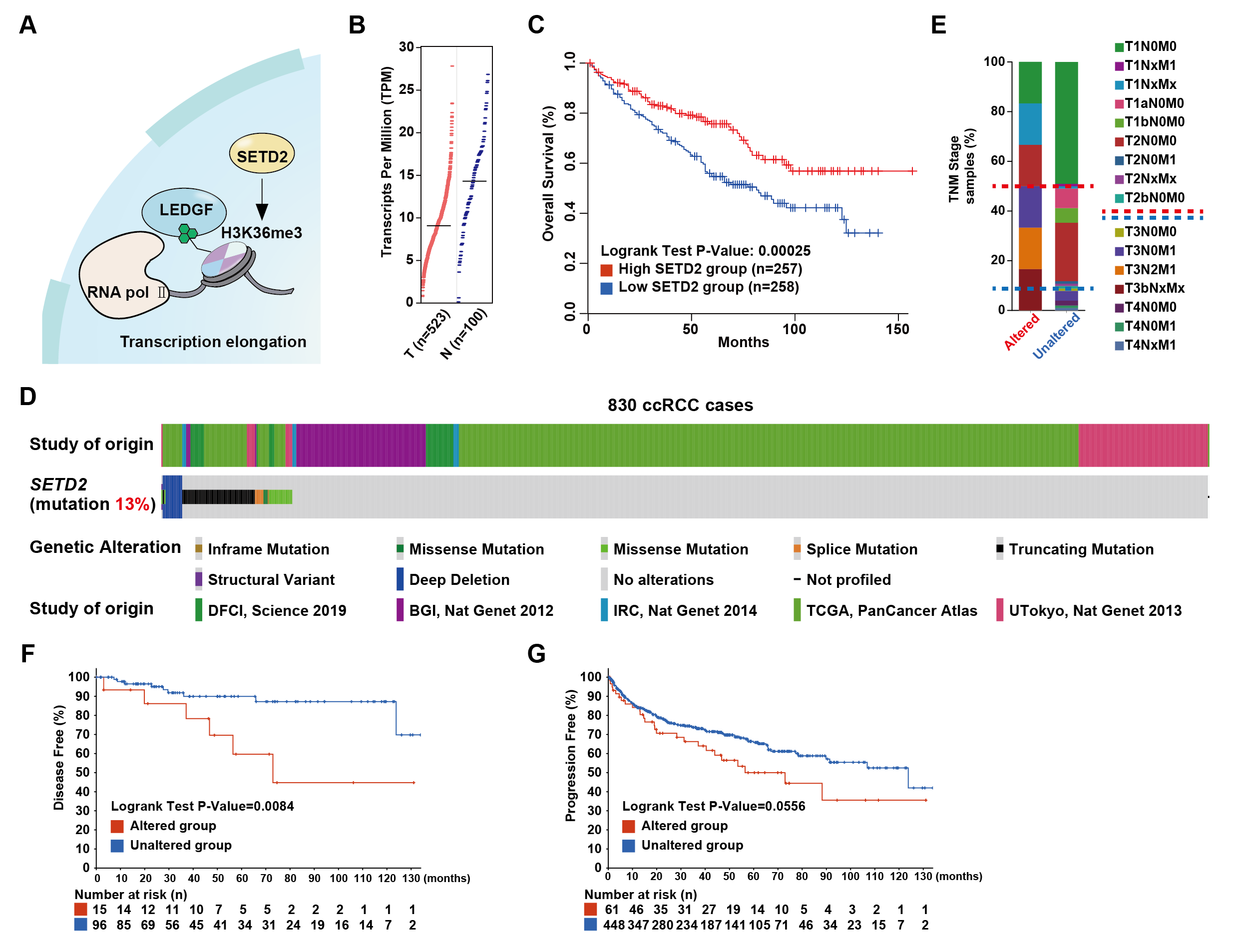


**Figure S2. High frequency of *SETD2* mutations in ccRCC patients is associated with poor prognosis.**

(A) A schematic model illustrating that LEDGF reads SETD2-dependent H3K36me3 mediating transcriptional elongation. (B) SETD2 mRNA expression was significantly down-regulated in ccRCC patients on the GEPIA database. (C) The overall survival of ccRCC patients with higher expression of SETD2 was longer on the GEPIA database. (D) *SETD2* exhibits a high frequency of mutations in ccRCC patients from the cBioPortal database. (E) The proportion of advanced patients in *SETD2-*mutant group was significantly higher from the cBioPortal database. The red line (*SETD2* altered) and the blue line (*SETD2* unaltered) are used to divide early T-stage (T1 and T2) patients and advanced T-stage (T3 and T4) patients. (F-G) *SETD2* mutation is associated with poor prognosis of ccRCC patients. *SETD2* non-mutated ccRCC patients showed longer disease-free (F) and progression-free survival (G) from the cBioPortal database.


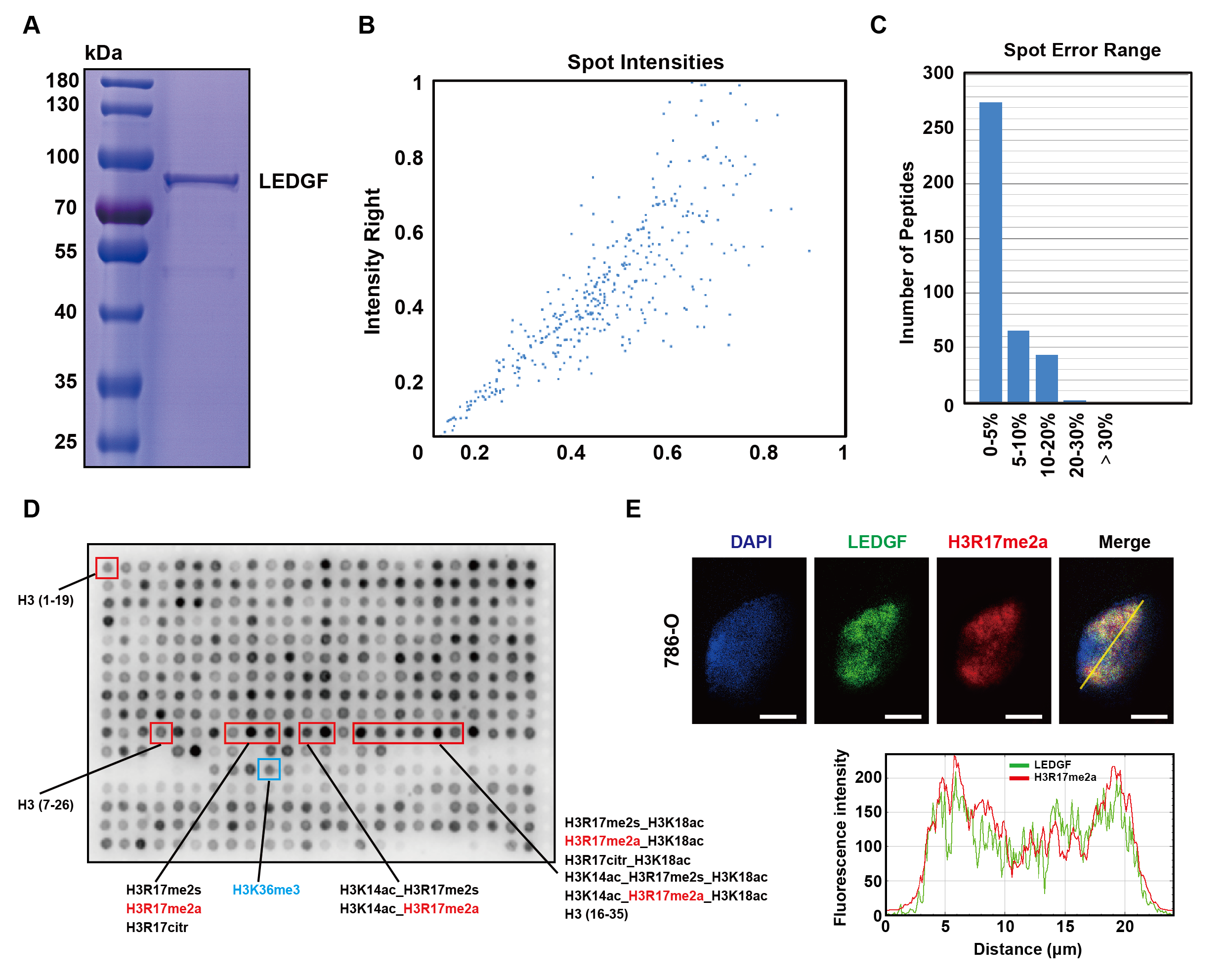


**Figure S3. LEDGF specially interacts with H3R17me2a.**

(A) LEDGF protein was successfully purified. (B-C) The quality inspection results of the modified Histone Peptide Array meet the standard using the Array Analyze Software (Active Motif). (D) Repeated experiment of co-incubation of LEDGF protein and the modified Histone Peptide Array shows the same trend. Red and blue boxes indicate specific modification marks. (E) Confocal microscopy images of immunofluorescence staining showing that LEDGF (green) colocalizes with H3R17me2a (red) in 786-O cells. Scale bar = 5µm.


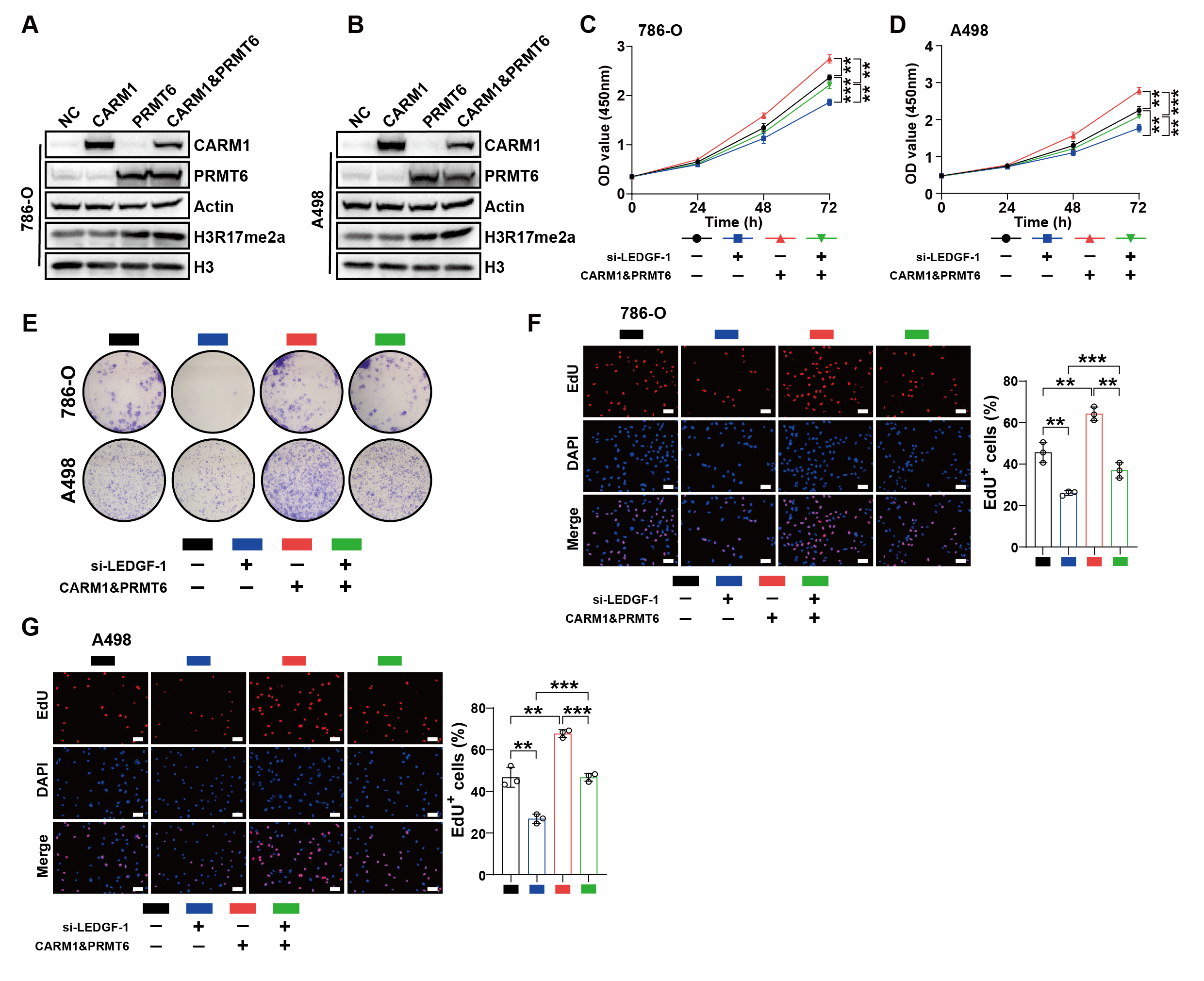


**Figure S4. Reduction of LEDGF can partially compensate for the cancer-promoting effect of increasing H3R17me2a level.**

(A-B) Western blot was performed to illustrate that simultaneous overexpression of CARM1 and PRMT6 significantly increased H3R17me2a level in 786-O cells (A) and A498 cells (B). (C-G) Reduction of LEDGF can partially compensate for the cancer-promoting effect of increasing H3R17me2a level in 786-O cells and A498 cells. CCK-8 assay (C-D), colony formation assay (E), and EdU proliferation assay (F-G) were performed to detect the proliferation ability of ccRCC cells. Scale bar = 100µm. Data are shown as mean ± SD. ***P* < 0.01, ****P* < 0.001.


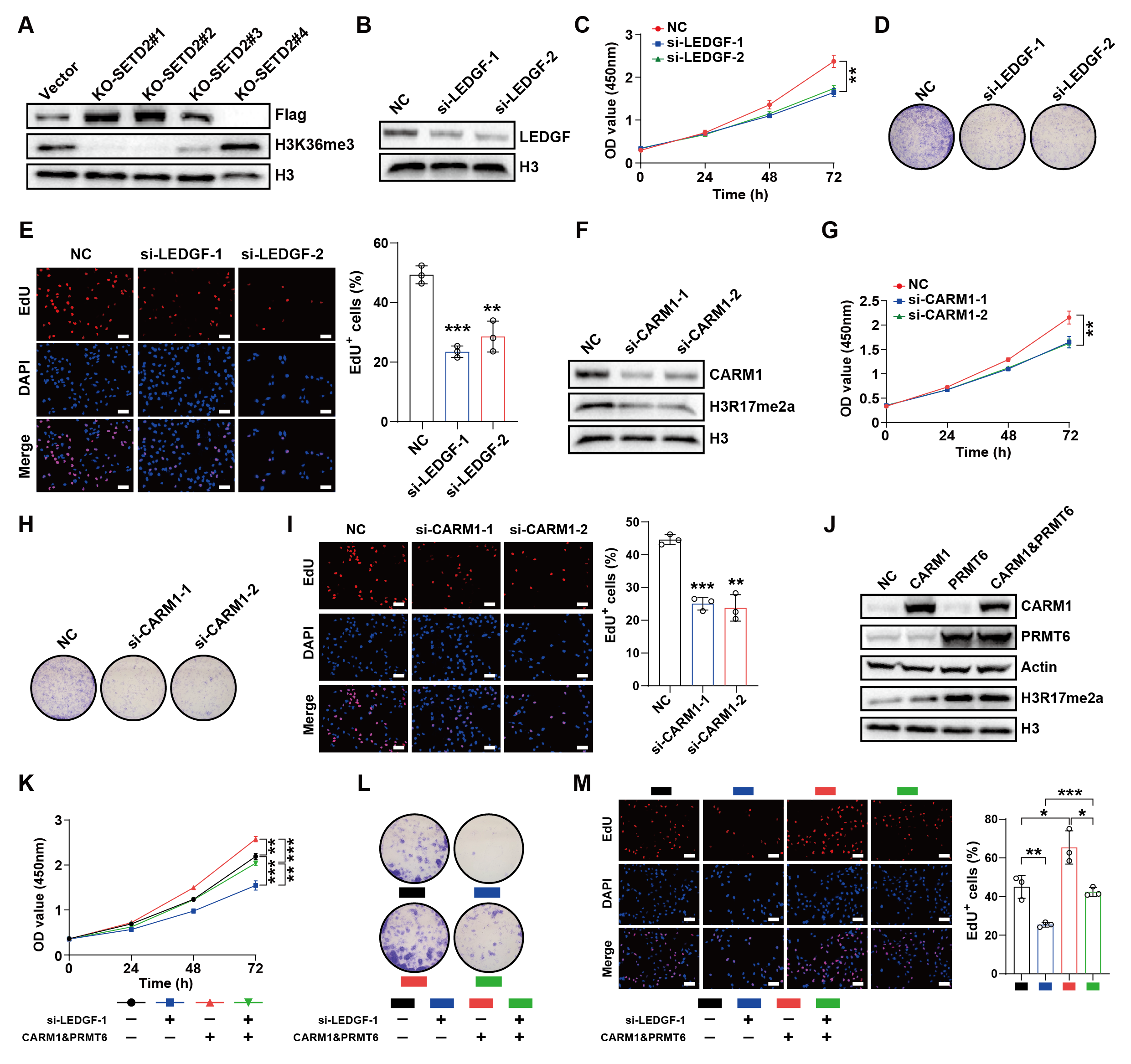


**Figure S5. H3K36me3-deficient cells showed similar changes to A498 cells.**

(A) Western blot of reduced H3K36me3 level in pooled 786-O cells transduced with SETD2-targeting CRISPR-Cas9 lentivirus. (B) The siRNA targeting LEDGF mRNA can effectively interfere with LEDGF expression in H3K36me3-deficient cells. (C-E) LEDGF knockdown significantly inhibited the proliferation of H3K36me3-deficient cells. CCK-8 assay (C), colony formation assay (D), and EdU proliferation assay (E) were performed to detect the proliferation ability after LEDGF knockdown. Scale bar = 100µm. (F) The siRNA targeting CARM1 mRNA can effectively interfere with CARM1 and decreases H3R17me2a in H3K36me3-deficient cells. (G-I) Reduction of CARM1 and H3R17me2a significantly inhibited the proliferation of H3K36me3-deficient cells, as demonstrated by CCK-8 (G), colony formation (H) and EdU proliferation assays (I). Scale bar = 100µm. (J) Western blot was performed to illustrate that simultaneous overexpression of CARM1 and PRMT6 significantly increased H3R17me2a level in H3K36me3-deficient cells. (K-M) Reduction of LEDGF can partially compensate for the cancer-promoting effect of increasing H3R17me2a level in H3K36me3-deficient cells. CCK-8 assay (K), colony formation assay (L), and EdU proliferation assay (M) were performed to detect the proliferation ability. Scale bar = 100µm. Data are shown as mean ± SD. **P* < 0.05, ***P* < 0.01, ****P* < 0.001.


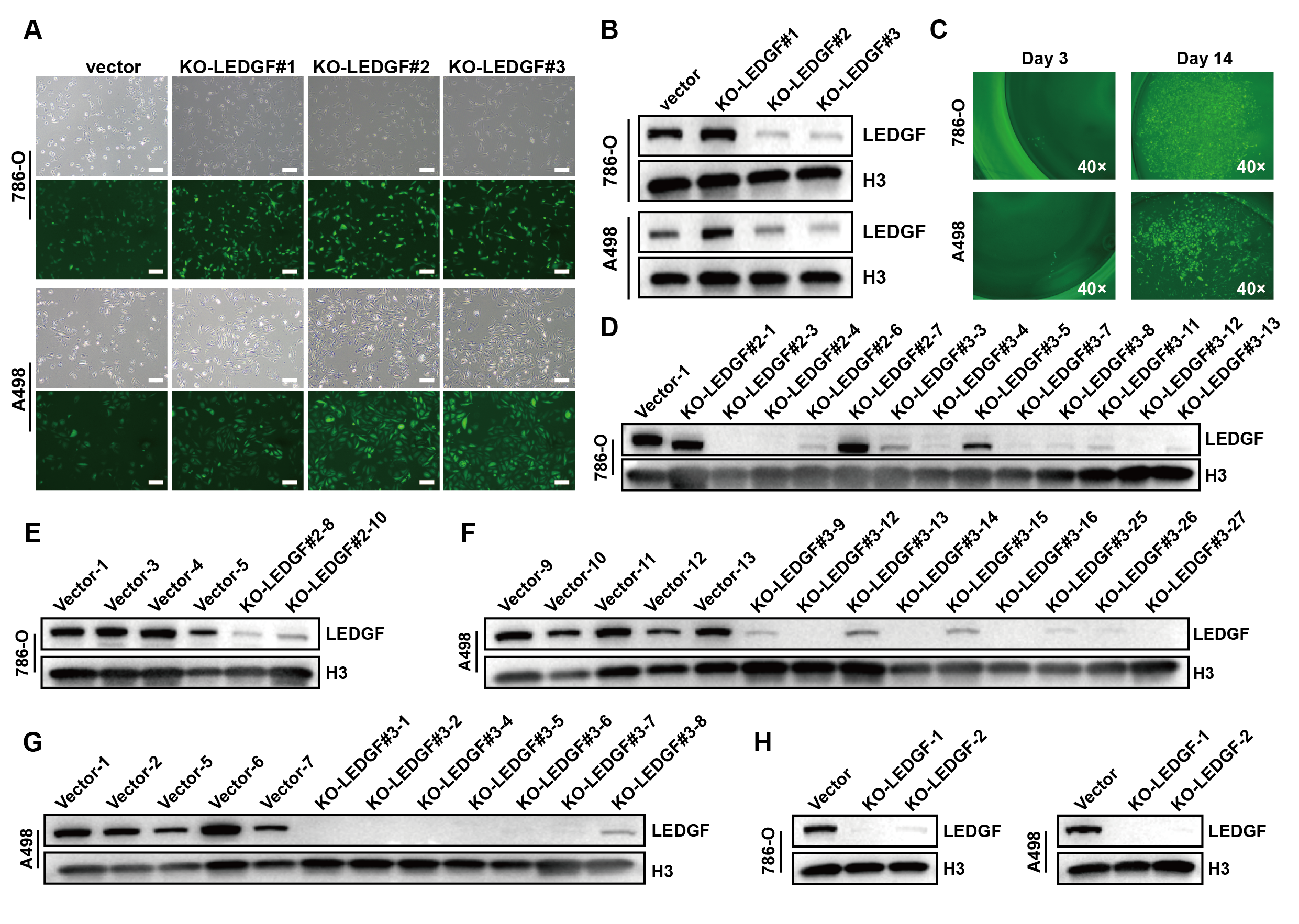


**Figure S6. Construction of LEDGF-KO cells.**

(A) The fluorescence images show the high infection efficiency of the LEDGF-targeting CRISPR-Cas9 lentivirus in ccRCC cells. Scale bar = 100µm. (B) Western blot of knockout efficiency of LEDGF in pooled ccRCC cells. (C) Culture of monoclonal ccRCC cells. The images were obtained at 40× magnification. (D-G) Western blot of knockout efficiency of LEDGF in monoclonal 786-O cells (D-E) and A498 cells (F-G). (H) LEDGF-KO cell lines were successfully constructed.


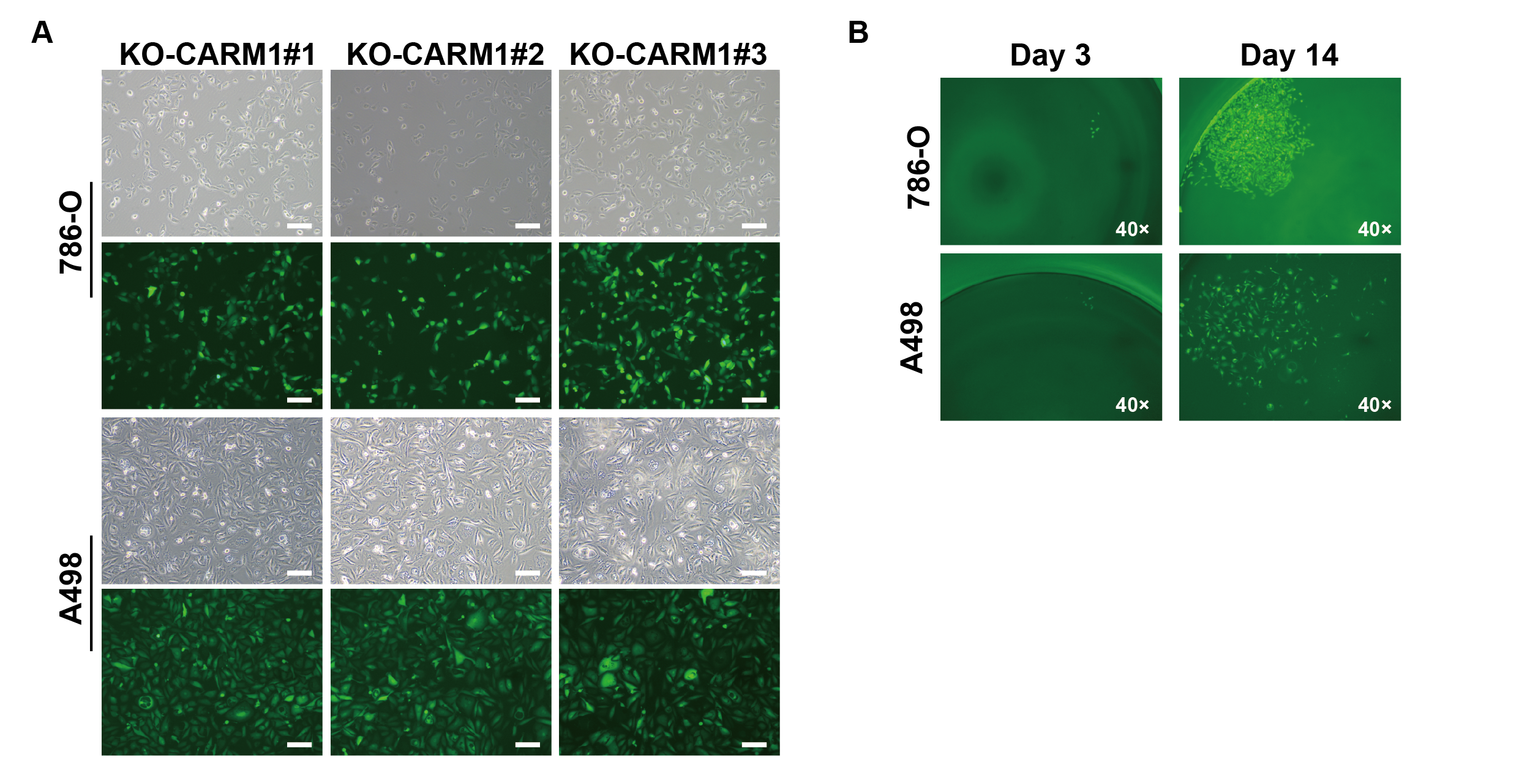


**Figure S7. Construction of CARM1-KO cells.**

(A) The fluorescence images show the high infection efficiency of the CARM1-targeting CRISPR-Cas9 lentivirus in ccRCC cells. Scale bar = 100µm. (B) Culture of monoclonal ccRCC cells. The images were obtained at 40× magnification.


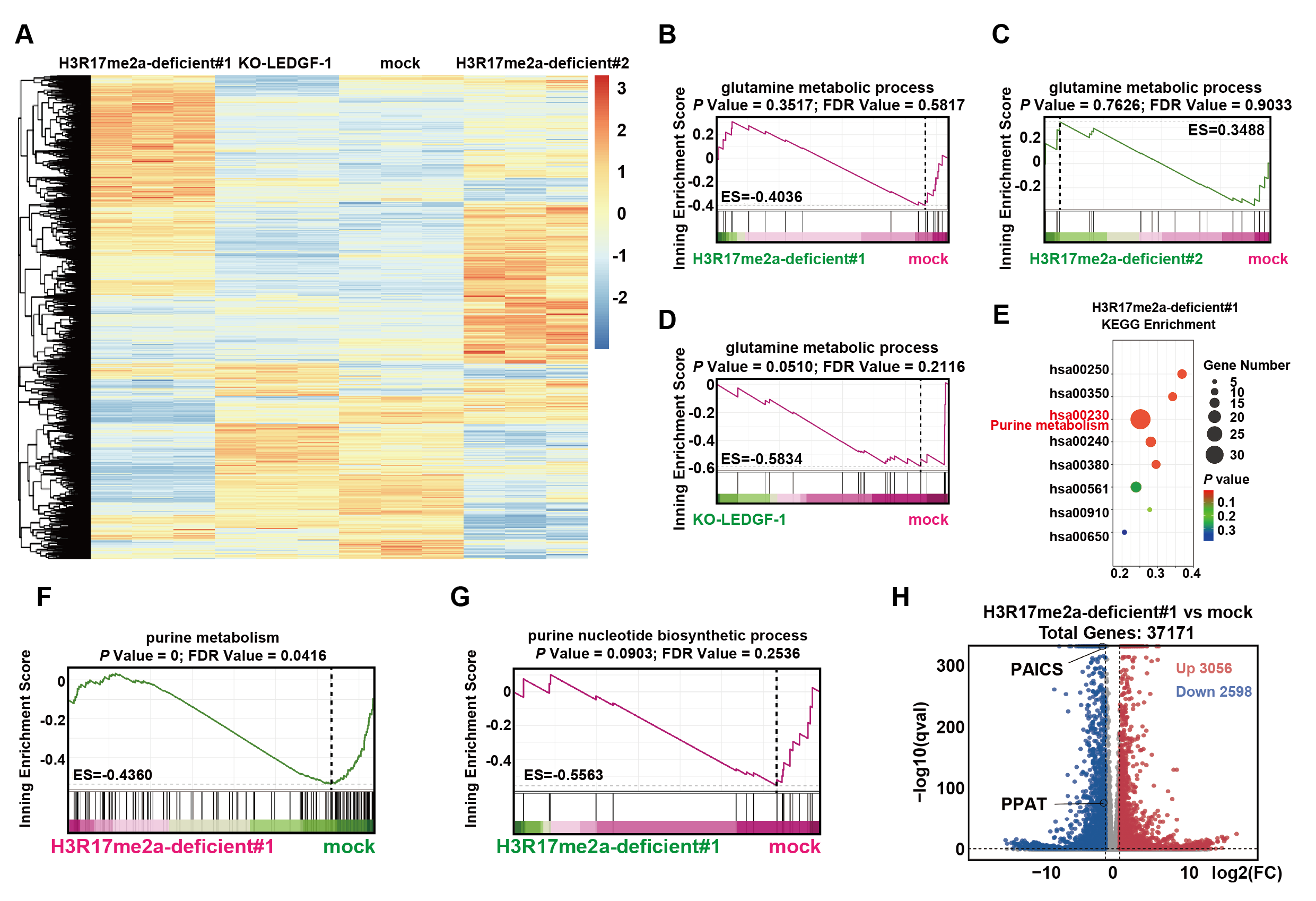


**Figure S8. RNA-sequence analysis of LEDGF-KO and H3R17me2a-deficient cells.**

(A) Heat map of different gene expression in indicated cells. (B-D) GSEA analysis of glutamine metabolic process in different groups. (E) KEGG enrichment analysis of differentially expressed genes (DEGs) in H3R17me2a-deficient#1 cells shows a close correlation with purine metabolism. (F-G) GSEA analysis of purine metabolism (F) and purine nucleotide biosynthetic process (G) in H3R17me2a-deficient#1 cells. (H) Volcano plots of DEGs in H3R17me2a-deficient#1 cells. Key enzymes in the de novo pathway are labeled such as PPAT and PAICS.


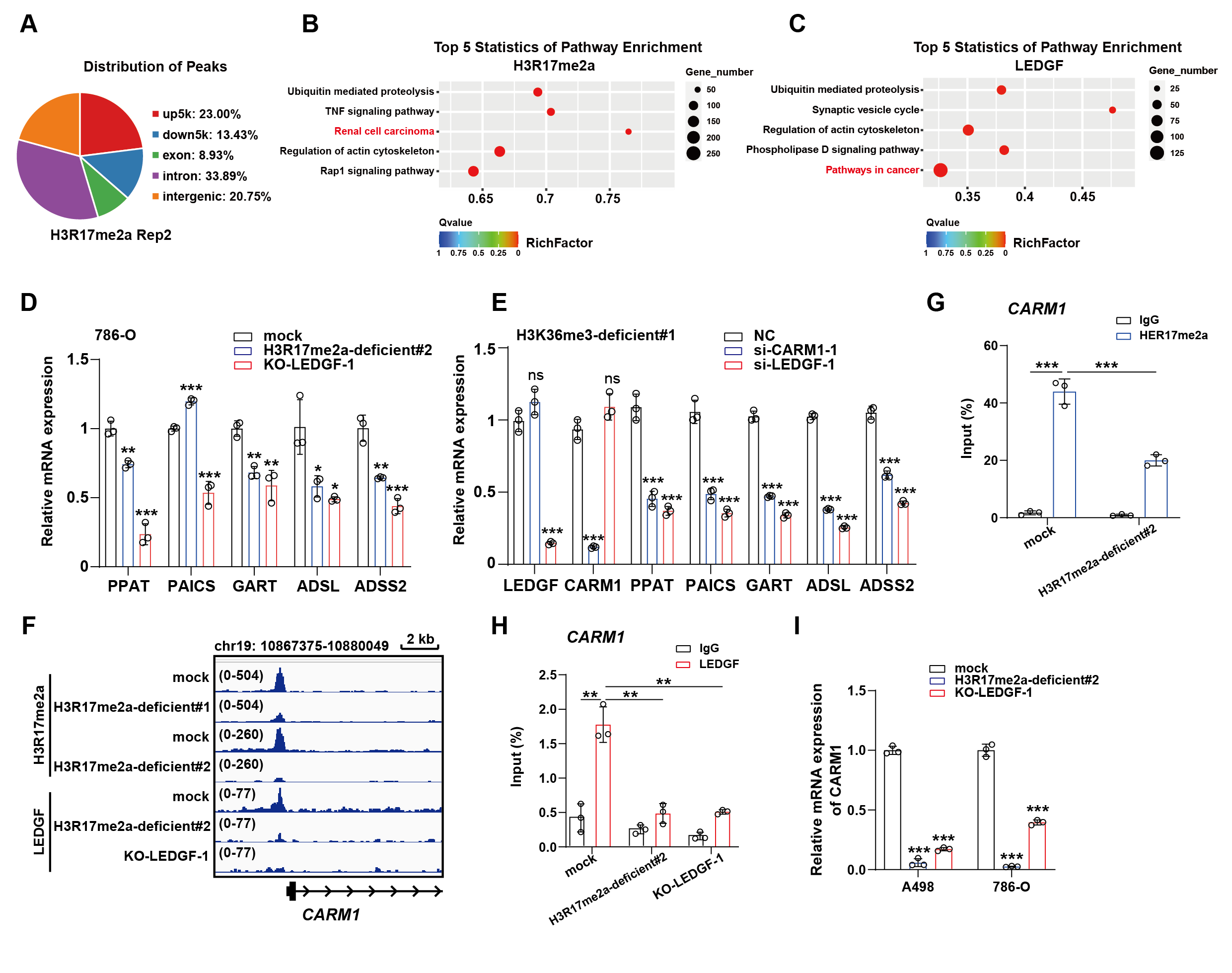


**Figure S9. LEDGF reads H3R17me2a regulating key enzymes in the de novo synthesis pathway.**

(A) Distribution of H3R17me2a peaks on the genome (repeated experiment). (B-C) Enrichment analysis showed that H3R17me2a and LEDGF were closely related to tumor. Only the top 5 pathway enrichment statistics are shown in the figure. (D) QRT-PCR assay demonstrated that LEDGF reads H3R17me2a regulating parts of key enzymes in the de novo synthesis pathway in 786-O cells. (E) Results of qRT-PCR illustrated that decrease of LEDGF or H3R17me2a level can reduce mRNA expression of key enzymes in the de novo synthesis pathway in H3K36me3-deficient#1 cells. (F) CUT&Tag profiles of H3R17me2a and LEDGF at the transcription start site (TSS) of *CARM1* in A498 cells. (G-H) The specific enrichment of H3R17me2a and LEDGF at the TSS of *CARM1* was verified by ChIP-qPCR assay. (I) QRT-PCR assays demonstrated that LEDGF reads H3R17me2a regulating CARM1 mRNA expression in 786-O and A498 cells. Data are shown as mean ± SD. **P* < 0.05, ***P* < 0.01, ****P* < 0.001.


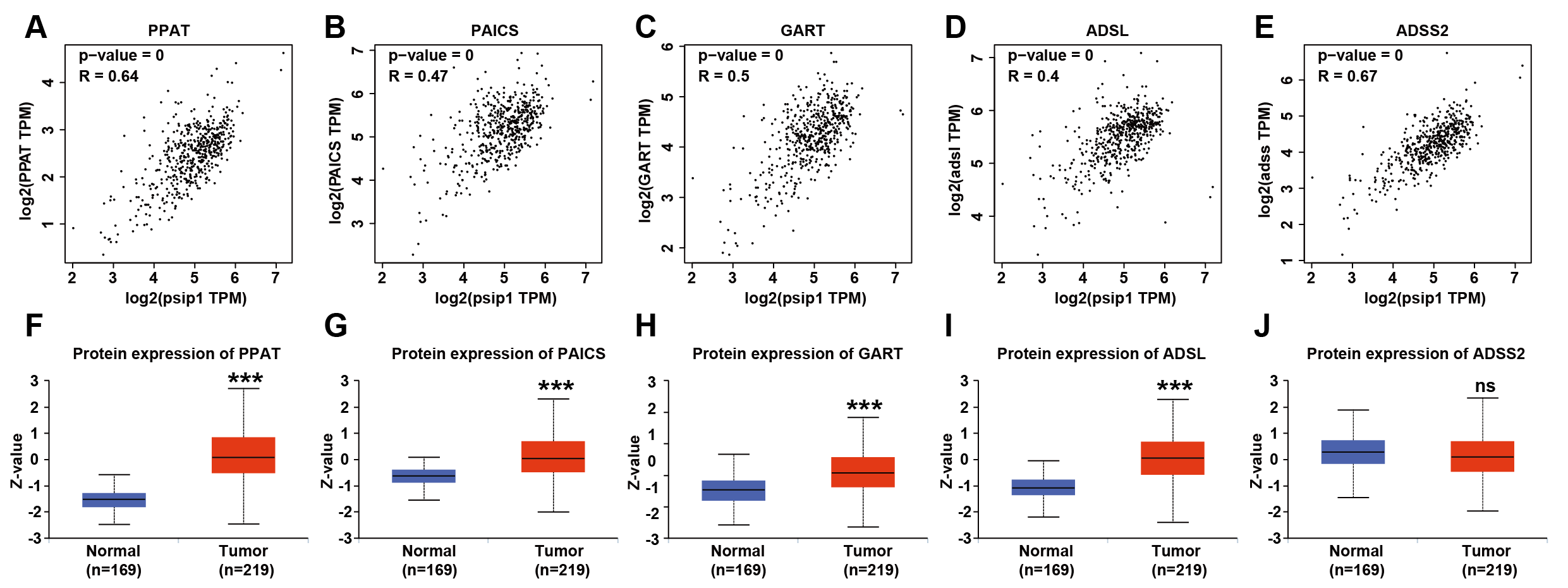


**Figure S10. Database information on key genes of the de novo synthesis pathway.**

(A-E) The mRNA expression of LEDGF was highly positively correlated with the key genes of de novo nucleotide synthesis on the GEPIA database. (F-J) The protein expression level of the key genes are higher in ccRCC on the UALCAN database, except ADSS2. Data are shown as mean ± SD. ****P* < 0.001. ns means no significance.
